# Quantitative Flavoprotein Fluorescence Parameters in Retinal and Optic Nerve Diseases: A Scoping Review

**DOI:** 10.3390/jcm15103942

**Published:** 2026-05-20

**Authors:** Gregorio Benites-Narcizo, Tamara Juvier-Riesgo, Adriana P. Pérez-Negrón, Luciana García-Dussán, Jianhua Wang, Jiang Hong, Carlos E. Mendoza-Santiesteban, Byron L. Lam

**Affiliations:** Bascom Palmer Eye Institute, University of Miami Health System, Miami, FL 33136, USA; txj362@miami.edu (T.J.-R.); app607@miami.edu (A.P.P.-N.); lxg1432@miami.edu (L.G.-D.); jwang3@miami.edu (J.W.); h.jiang@miami.edu (J.H.); cmendoza@miami.edu (C.E.M.-S.); blam@miami.edu (B.L.L.)

**Keywords:** flavoproteins, mitochondrial fluorescence, metabolic imaging, retinal diseases, retinal degeneration, retinal dystrophies, optic nerve diseases

## Abstract

**Background**: Retinal and optic nerve disorders remain major causes of visual morbidity worldwide. Ocular fundus flavoprotein fluorescence (FPF) imaging has emerged as a potential noninvasive biomarker of mitochondrial dysfunction for earlier detection and evaluation of disease severity. **Methods**: We conducted a Systematic Scoping Review of the diagnostic and correlational utility of quantitative FPF parameters in retinal and optic nerve diseases compared with healthy controls. Following PRISMA-ScR guidelines, we searched MEDLINE, Web of Science, Scopus, and CENTRAL for peer-reviewed human studies available online before 31 December 2025. **Results**: Seventeen studies were included, encompassing 1914 eyes and 1339 participants, and were predominantly cross-sectional. In healthy eyes, mean macular and optic nerve head FPF intensity were reported as 24.1 ± 12.2 gsu and 30.6 ± 14.6 gsu, respectively. Higher signals were reported in several disorders, including diabetes mellitus (76.0 [67.0–92.0] gsu), neovascular age-related macular degeneration (67.47 ± 17.77 gsu), and retinitis pigmentosa (50.5 ± 12.2 gsu). However, lower, unchanged, or stage-dependent signals were also observed within the same disease categories. Interpretation across studies was limited by substantial heterogeneity in patient selection, disease definitions, imaging protocols, control groups, and FPF outcome metrics. The precise cellular and sublayer origin of the detected signal also remains challenging to determine. **Conclusions**: Ocular fundus FPF imaging provides promising metabolic insight into retinal and optic nerve diseases. However, current evidence remains heterogeneous and largely cross-sectional, limiting clinical interpretability and generalizability. Longitudinal studies, technical standardization, and multimodal integration are needed to define reproducible disease-specific FPF profiles and improve translational applicability.

## 1. Introduction

Retinal and optic nerve diseases impose substantial visual morbidity worldwide, representing the leading causes of irreversible blindness [1]. In 2021, the global age-standardized prevalence of blindness was 15,784 per 100,000 population, with glaucoma, age-related macular degeneration (AMD), cataract, and diabetic retinopathy (DR) among the major contributors [2]. The global disability-adjusted life years attributable to blindness and vision loss increased from 12.44 million in 1990 to 22.56 million in 2019, with projections indicating continued growth as populations age [3]. Earlier detection of cellular stress in these conditions could significantly improve monitoring and treatment decisions, as many causes of vision impairment can be prevented or treated if identified before irreversible structural damage occurs.

Emerging technologies are being developed to address retinal and optic nerve diseases. Artificial intelligence and deep learning systems have demonstrated high accuracy in screening for DR, AMD, glaucomatous optic neuropathy, and other abnormalities using fundus images [4]. Most current imaging analysis of the eye fundus is structural rather than metabolic. While optical coherence tomography (OCT), fundus photography, fundus autofluorescence (FAF), and fluorescein angiography provide detailed anatomical information, they primarily detect disease after structural damage has already occurred [5].

There is growing recognition that metabolic dysfunction often precedes structural damage in retinal diseases, creating a critical window for early intervention that structural imaging alone cannot capture [6,7].

Several eye imagers used for metabolic assessment are adaptations of existing fundus autofluorescence and laser scanning ophthalmoscopy (SLO) technologies, which rely on detecting light from excited fluorophores and reflected or emitted light from the posterior pole, respectively [8,9,10]. Such modifications lead to integrated devices, including Adaptive Optics Scanning Laser Ophthalmoscopy (AOSLO), Adaptive Optics Near-Confocal Ophthalmoscopy (AONCO), and Adaptive Optics Rolling Slit Ophthalmoscopy (AO-RSO) [8,11,12,13]. Other functional imaging techniques, such as multifocal electroretinography, microperimetry, and dark adaptometry, have evaluated retinal function with limited resolution [11].

Flavoprotein fluorescence (FPF) imaging is emerging as a tool for metabolic imaging of the eye by applying a similar principle: oxidized flavoproteins absorb blue light and emit green autofluorescence. Several studies have demonstrated clinical utility across multiple retinal and optic nerve diseases. As a functional mitochondrial imaging tool, FPF imaging shows promise for evaluating retinal and optic nerve conditions and may enable earlier detection and intervention than conventional approaches [6,12,13,14].

Despite growing interest in FPF as a potential noninvasive biomarker of mitochondrial dysfunction, several difficulties have been encountered in reaching consensus on patient profiles, standardized metrics across studies, and clinically significant results. The evidence remains fragmented across conditions, with variability in acquisition methods, reported metrics (e.g., intensity/heterogeneity), and the comparator selection (OCT, FAF, visual field, visual acuity), limiting synthesis and clinical interpretability [12,15,16].

In this context, we conducted a Systematic Scoping Review of the diagnostic and correlational utility of quantitative FPF parameters in patients with retinal or optic nerve diseases, compared with healthy controls, for detecting damage or disease severity. We aimed to contribute to a clear understanding of the current advances in the field, which could aid in guiding future research. Additionally, a comprehensive literature review of the technique’s key insights was conducted.

## 2. Materials and Methods

We conducted a literature review in the MEDLINE via Ovid (Ovid Technologies, New York, NY, USA), Web of Science Core Collection (Clarivate, London, UK), Scopus (Elsevier B.V., Amsterdam, The Netherlands), and CENTRAL (Cochrane Central Register of Controlled Trials; Cochrane, London, UK) databases to identify reports on FPF in the fundus of the eye. A research algorithm was designed based on the following Medical Subject Headings (MeSH) and free-text terms related to: ‘Flavoproteins’, ‘FPF imaging’, ‘redox imaging’, ‘Mitochondrial fluorescence’, ‘Retinal Degeneration’, ‘Optic Nerve Diseases’, ‘Macular Degeneration’, ‘Diabetic Retinopathy’, ‘Glaucoma’, ‘Central serous chorioretinopathy’, ‘Stargardt disease‘, ‘Leber congenital amaurosis’, ‘Leber hereditary optic neuropathy’, ‘optic atrophy’, and ‘metabolic imaging’ (See Supplementary Table S1 for search strategy). All included articles were available online before 31 December 2025. Potentially selectable works were retrieved by two researchers (A.P.P.-N. and L.G.-D.) and discussed by a third researcher (G.B.-N.). All the results included in this Systematic Scoping Review were reported following the Tricco et al. (2018) PRISMA Extension for Scoping Reviews (PRISMA-ScR) guidelines (See Supplementary Table S2 for both the extended PRISMA and Scoping Review checklists) [17]. We did not publish a PROSPERO protocol.

### 2.1. Inclusion and Exclusion Criteria

The following inclusion criteria were considered to select the papers for further analysis:

- Original research studies published in peer-reviewed journals in English.
- Studies that include human participants of any age or sex with retinal and optic nerve diseases who have undergone fundus flavoprotein fluorescence imaging.
- Studies that assess healthy human participants who underwent fundus flavoprotein fluorescence imaging.

We excluded articles written in a language other than English, reviews and meta-analyses, editorials and commentaries, conference abstracts without full data, unpublished dissertations or non-peer-reviewed reports, animal studies, in vitro (laboratory-based) studies, and studies where flavoprotein fluorescence was measured in ocular tissues other than the retina or optic nerve (i.e., not fundus imaging).

### 2.2. Data Extraction

Two independent reviewers (A.P.P.-N. and L.G.-D.) extracted data from the selected reports, and the findings were corroborated by a third reviewer (G.B.-N.). A title and abstract search was conducted, and the results were saved in EndNote Reference Manager. All data was collected and summarized in tables using Microsoft Office Excel 365 using the following standardized data extraction form:

- Study characteristics: Authors, publication year, country, study design, setting.
- Population details: Sample size, age, sex, health status (specific disease details).
- Intervention details: Device, pre-procedure preparation, technique utilized, and specific FPF measurement parameters considered.
- Comparator details: Device and measurement parameters considered for established techniques (Fundus autofluorescence or FAF/Optical coherence tomography or OCT), FPF values in healthy controls.
- Primary and secondary outcomes: intensity, heterogeneity, correlation between healthy and diseased patients, correlation between FPF and standard techniques (FAF or OCT), normality profile depending on age and sex.
- Results and effect estimates: means, standard deviations, risk ratios, odds ratios, confidence intervals.
- Funding source and conflict of interest declarations

### 2.3. Quality Assessment

We did not perform a quality assessment of the articles due to the heterogeneity and scarce availability of reports for this scoping review.

### 2.4. Data Synthesis

A narrative review of the selected studies was conducted due to their heterogeneity in approach. FPF intensity was the primary metric analyzed. When possible, FPF heterogeneity was included, along with correlations between FPF and other diagnostic images, such as OCT, FAF, or best corrected visual acuity (BCVA).

Not all studies compared healthy patients with patients with cases, and several developed their own algorithms for image analysis. Particular results were selected to be described and discussed.

### 2.5. Data Analysis

Comparisons between groups in the studies were analyzed using descriptive statistics. The outcome variables were grouped by disease, and, where required, equivalence was estimated to homogenize the available data. Direct calculations were performed using the data available in the paper or, when possible, the supplementary data.

The principal outcome metrics considered were *p*-values and 95% confidence intervals. When available, other metrics were considered: mean difference or standardized mean difference, confidence intervals, sensitivity, specificity, area under the curve (AUC), Correlation coefficients, Spearman’s ρ (rho) values, Pearson correlation coefficient, R^2^ values (Coefficient of Determination from regression).

## 3. Results

### 3.1. Study Selection

**Figure 1:**
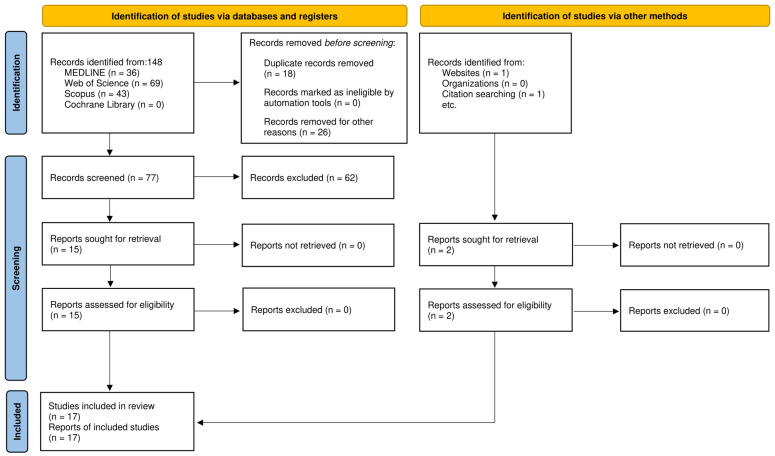
PRISMA flow chart diagram.

Seventeen studies from 148 preselected records were included in the final analysis, comprising data from 1914 eyes and 1339 subjects, including patients and healthy controls. See Figure 1 for details in the PRISMA flow diagram for the study selection process.

### 3.2. Study Features

The majority of the studies (15) were conducted using a cross-sectional design, and the remaining (two) used a comparative case-series design. A total of 700 patients, 639 controls, and 1914 total eyes were studied using FPF since the first report in 2008 [18]. Most were performed in the USA [12,13,14,15,16,18,19,20,21,22,23,24,25,26,27,28], and only one work was executed in Germany [6]. Eleven reports included both patients’ eyes in their cohorts and treated them independently [6,12,13,16,18,21,22,25,26,27,28]; only one used the mean value from both eyes [15]. Two cohorts included only cases [20], and one included only healthy people [15]. See Table 1 and Table 2, and Supplementary Table S3 for further details on demographic and design data. The main outcomes of the study are described and discussed in Section 4.4 and Section 4.6.

## 4. Flavoprotein Fluorescence and Eye Diseases

### 4.1. Flavoproteins and Mitochondrial Metabolism

#### 4.1.1. Mitochondria in the Retina and Optic Nerve

The retina consumes more oxygen than any other organ in the body, with the photoreceptor inner segments having the highest mitochondrial concentration and activity. Due to this and the high concentration of polyunsaturated fatty acids in the outer retinal segments, these are highly susceptible to oxidative damage and lipid oxidation [29].

There are few reports on the actual mitochondrial distribution or percentage concentration across retinal layers; however, some authors have provided a general overview of how it occurs (See Table 3). Chidlow et al. demonstrated across vascularized mammalian retinas, including those most comparable to humans, that mitochondria are most abundant in photoreceptor inner segments, the plexiform layers, retinal ganglion cells, and the basolateral surface of the retinal pigment epithelium [30]. Those segments exhibited the strongest labeling and the highest enzymatic activity of cytochrome c oxidase, succinate dehydrogenase, and isocitrate dehydrogenase, confirming their status as the principal mitochondrial reservoir in the outer retina. In the inner retina, both the inner and outer plexiform layers showed intense mitochondrial protein expression and activity in vascular species, reflecting the substantial energetic demand of neurotransmission at ribbon synapses. Retinal ganglion cells also consistently displayed dense mitochondrial labeling, supporting their reliance on oxidative phosphorylation for unmyelinated axonal conduction [30].

While Chidlow et al. provided essential layer-level insights into mitochondrial distribution, recent human ultrastructural work by Kar et al. revealed a far more intricate organization specifically within the outer plexiform layer (OPL) and adjacent inner nuclear layer (INL) [31]. Using volumetric electron microscopy and deep learning reconstruction, they found that the human OPL consists of multiple discrete mitochondrial sublayers rather than a single homogeneous band. These sublayers correspond to mitochondria clustered within cone and rod terminals (OPL1a), a mitochondria-sparse zone between pedicles (OPL1b), and mitochondria-rich regions formed by dendrites of bipolar and horizontal cells (OPL2a, OPL2b). The highest mitochondrial occupancy in this region was observed in a distinct sublayer at the INL–OPL border (INLm), which corresponds to dense postsynaptic dendritic architecture [31].

Inside retinal ganglion cells (RGCs), mitochondria are thought to follow a mix of static and bidirectional movement from one cellular pole to another. They use cellular microtubular kinesins for anterograde movement and dynein for retrograde movement; this movement can be altered by several conditions, like cellular stress, glucose levels, or calcium signaling [32,33]. Additionally, these organelles exert a dynamic cycle of fission and fusion depending on the metabolic status [34].

Taken together, mitochondria are strategically concentrated in the retina’s most metabolically active cell types, where they provide essential energy for phototransduction, synaptic transmission, and cellular homeostasis [35].

Mitochondrial density is estimated to be 16,000 to 21,000 per mm^2^ in the area behind the ONH [36]. This is thought to be the highest concentration found in RGC axons, especially in the laminar and prelaminar regions, as well as at the myelin transition zone (MTZ), likely to equilibrate the elevated concentration of ion channels in these unmyelinated axonal zones [21,34]. Approximately three-quarters of mitochondria tend to stay stationary in their designated area of metabolic demand. The rest is divided into two groups: one moves slowly towards the brain, and the other moves faster in the opposite direction; they are thought to return to the soma to restore components and functions [36]. The MTZ has also been identified as the site of mitochondrial clearance in RGCs [34]. In the unmyelinated regions of RGC axons in the inner retina, mitochondria are uniformly distributed [37].

Because mitochondrial failure precedes structural degeneration, the optic fundus offers a unique in vivo window into retinal bioenergetics [14]. Alterations in mitochondrial function, whether through impaired mitophagy, disrupted fusion–fission balance, or lysosomal degradation defects, can lead to early metabolic stress detectable before overt clinical changes [35]. This has positioned mitochondrial imaging and functional biomarkers as emerging tools for identifying subclinical disease activity and understanding the pathophysiology of conditions such as primary open-angle glaucoma (POAG), diabetic retinopathy (DR), retinitis pigmentosa (RP), and age-related macular degeneration (AMD).

#### 4.1.2. Flavoproteins: Structure, Function, and Fluorescence

Flavoproteins, more commonly enzymes, work with a flavin cofactor, such as flavin mononucleotide (FMN) or flavin adenine dinucleotide (FAD), which comes from enzymatic modification of dietary riboflavin [38]. These cofactors confer redox reactivity through their isoalloxazine ring system, which can exist in three oxidation states: fully oxidized (quinone), one-electron reduced (semiquinone), and fully reduced (hydroquinone) [39]. Because of that property, flavoproteins primarily catalyze oxidation–reduction reactions. They are central to core metabolic processes, including the citric acid cycle-related reactions, the mitochondrial electron transport chain, β-oxidation, and amino acid degradation [38].

At the mitochondrial level, FAD oxidation is among the earliest indicators of damage, occurring before the onset of disease-related cell death [18]. Given this, flavoproteins in the electron transport chain are oxidized and emit green autofluorescence (peak ~520–540 nm) upon excitation with blue light (~430–470 nm), enabling non-invasive functional imaging [14]. Even in the basal state, mitochondrial flavoprotein oxidation results in this phenomenon [23,40]. This FPF originates from the previously mentioned prosthetic groups, FAD or FMN. Approximately 75% of the FAD fluorescence observed in mammalian mitochondria arises from flavoproteins such as lipoamide dehydrogenase and the electron transfer flavoprotein (ETF) [28,41]. Conversely, mitochondrial inner membrane complexes I and II are more involved in this phenomenon [42,43,44].

When previously reduced cofactor-containing molecules become oxidized under an unstable or knocked-down mitochondrial membrane potential (ΔΨm), especially during proapoptotic conditions, the electrons within the co-factor isoalloxazine ring enter a highly resonant state, therefore becoming sensitive enough to undergo a superior energetic state (singlet state or S_1_), induced by the blue light beam’s brief excitation [25,28,41]. Finally, the electrons undergo a reverse energy transition to lower orbitals (the ground state or S_0_), emitting green light [28]. In dead cells or those experiencing the final stages of cell death, this fluorescence disappears [24]. Nevertheless, the resultant signal also depends on the protein microenvironment; not all FAD-containing proteins in mitochondria are bright to the same extent, or at all. It was found that, in other proteins such as succinate dehydrogenase, FAD can be quenched by aromatic residues (e.g., tryptophan and tyrosine), thereby shortening the fluorescence lifetime and reducing emission intensity [28,41].

### 4.2. Historical Perspective

#### 4.2.1. Discovering the Phenomena and Early Animal Studies

Britton Chance first described FPF in 1955, showing that cell autofluorescence comes from mitochondrial flavoproteins and is related to their redox state [40,45,46]. Subsequent studies reported green light at 520–540 nm from various animal tissues upon excitation with blue light at 460 nm, consistent with the spectrum of flavoproteins [47,48]. Signal intensity or appearance changed upon different types of stimuli, like electric stimulation in the brain cortex (Rosenthal et al. 1971 [49]); redox reactions in neurons (Benson et al. 1979 [50]), skeletal (Kuznetsov et al. 1998 [51]) and cardiac myocytes (Romashko et al. 1998 [52]); and hypoxia in myocytes (Koke et al. 1981 [53]).

Others consequently identified the exact proteins from which the fluorescence came. They identified α-lipoamide dehydrogenase, ETF, and acyl-CoA dehydrogenase as the primary fluorescent proteins in their oxidized form [52,54]. In vivo studies, such as that by Shiino et al. (1998 [55]), reported that the hippocampus of Mongolian gerbils subjected to transient forebrain ischemia showed poor recovery of the mitochondrial FPF signal. Suggesting that mitochondrial redox imbalance and electron transport dysfunction are linked to delayed neuronal death. Others, such as Shibuki et al. (2003 [56]), confirmed that the FPF signal increases in vivo following electrical neural stimulation. Reinert et al. (2004 [57]) found that mitochondrial FPF correlated with neuronal activity in the mouse cerebellum, exhibiting a biphasic fluorescence pattern. Between 2007 and 2011, they further showed that this biphasic signal comprises a light phase, driven by flavoprotein oxidation and mainly reflecting neuronal oxidative metabolism, and a dark phase, driven by flavoprotein reduction and associated with glial glycolysis and lactate production [58,59].

Other researchers continued to increase the FPF signal in studies of in vivo ischemia in heart tissue (Ranji et al., 2006 [60]). Then the technique was introduced to map the cerebral cortex and to identify a spatiotemporal profile (Husson et al., 2007 [61]), plasticity in sensory regions (Tohmi et al., 2009 [62]), and highly oxygen-dependent regions (Chisholm et al., 2016 [63]).

#### 4.2.2. First Human Pilot Studies

Winkler et al. (1995 [64]) investigated FPF in saponin-skinned human myocytes with chronic progressive external ophthalmoplegia. They found that cells, particularly those with multiple mitochondrial DNA mutations, exhibited impaired mitochondrial redox dynamics. Kunz et al. (1997 [65]) and Kunz et al. (2002 [66]) found a higher FPF in human mononuclear cells, mainly due to electron-transfer flavoprotein α-lipoamide dehydrogenase in its oxidized state, compared to healthy cells.

From there, several human tissues have been studied using FPF. Wiedemann et al. (1998 [43]) reported elevated FPF levels in skeletal muscle in sporadic amyotrophic lateral sclerosis, even after the addition of reducing or oxidizing agents. In individual cells, higher mitochondrial FPF signals were found in the oxidized state across human myocytes, fibroblasts, blood mononuclear cells, peripheral blood monocytes, and neutrophils. It was also found that flavoprotein intensity can oscillate over time. It was also found that two intracellular FPF pools exist: a diffuse mitochondrial component and a peripheral punctate component linked to the NADPH oxidase flavoprotein [67].

Schweitzer et al. (2002 [68]) pioneered the transition to imaging the optical fundus using time-resolved autofluorescence with a laser-scanning ophthalmoscope, enabling measurement of lifetime distributions of various retinal fluorophores. They identified free flavins with a lifetime of approximately 2.0 ns, distinct from compounds like lipofuscin.

As the eye is approached, Susan Elner et al. (2008 [28]) evaluated FPF in cultured human retinal pigment epithelial cells. They found a marked increase during induced apoptosis, which was mitigated by antioxidant agents. Field et al. (2011 [69]) found a rapid, significant rise in FPF in cultured human and rat RPE cells exposed to varying levels of oxidative stress, correlating with ΔΨm loss and apoptosis within 1–2 h. These effects were reduced or blocked by antioxidants, indicating oxidative damage drives mitochondrial changes. This ultimately moved the attention to the possibility of analyzing eye structures.

Finally, in 2008, Victor Elner and colleagues developed the fundus camera-based technique to capture fluorescence lifetime imaging in vivo in human retinas. They observed elevated metrics in patients with conditions such as diabetes, AMD, CSR, and retinal dystrophies, compared to healthy controls [18,27,28]. Since then, several studies have been conducted to uncover new approaches to mitochondrial metabolic features at the optic fundus.

### 4.3. Flavoprotein Fluorescence Imager

**Figure 2:**
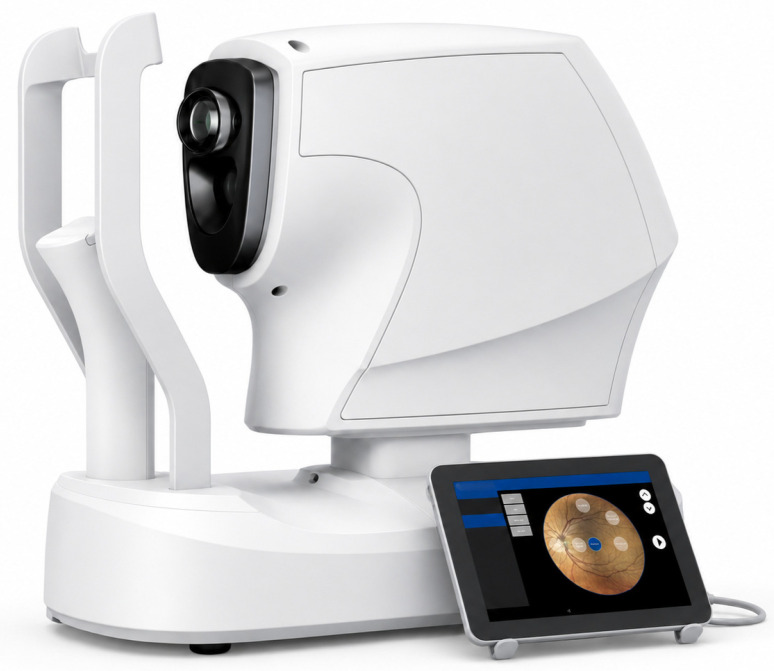
Third-generation eye fundus–flavoprotein fluorescence-capture device [71].

Three generations of FPF trackers were developed [23]. This device (OcuMet Beacon, OcuSciences Inc., Ann Arbor, MI) (See Figure 2) is a confocal, LED-based ophthalmoscope that uses infrared (IR) and blue light spectral regions to generate retinal images [70]. The excitation blue light source is a blue LED with a peak output at ~450–470 nm that delivers a brief flash of light for approximately 1 millisecond to excite the flavoproteins while protecting the eye from phototoxicity [19,25,27,70]. Another IR LED at 860 nm was added in the second-generation devices after 2012 [25], which used a scanning laser ophthalmoscope. The aim was to obtain reflectance images and to align the structures [25,71]. The confocal design, combined with the infrared alignment channel, helps suppress background autofluorescence from anterior structures such as the lens or cornea [23,70].

Currently, this machine is classified as a Type I safe laser and light-emitting diode by the International Electrotechnical Commission guidelines (ANSI Z80.36-2016 and ISO 15004-2:2007) [70]. The blue illumination is nearly 100 times below the international maximum permissible exposure limits, which is lower than that of a standard fundus camera [16,25].

Early versions used two Photometrics 512B back-illuminated, electron-multiplying charge-coupled device cameras, which were cooled to −30 °C to reduce noise, and attached to another Zeiss F4 camera with 512 × 512-pixel resolution, owing to their significant sensitivity to low-light signals; they both captured a 3° field of view for FPF [18,27,28]. It improved to 15° for FPF and to 30° for IR in the second generation. Those changes enhanced the device’s ability to track FPF signals and reduced fluorescence from other eye structures [19,23,25].

The third-generation device camera was a confocal infrared scanner that expanded the field of view to 23° for FPF and 60° for IR and captured approximately 5 megapixels (120 × 1656) with low noise [23,71]. The infrared images in the last device, version 1.6 (k241931) registered in the FDA database, are captured with a field of view (FOV) of 60° (H) × 21.5° (V), while the FPF captures are 17° (H) × 21.5° (V) [70].

Emission and detection filters for 460–475 nm and 520–540 nm, respectively, have been included since the prototype [18,23,70]. They included a dichroic filter to direct the incoming and emerging light beams, and an optical mask in the subsequent design. In the third generation, the camera had band-pass filters to reduce contamination from different fluorophores [23].

The images are received in grayscale, with the FPF ones being pseudo-colored for later analysis [71]. In the prototype, once the image was captured, it was processed using customized image analysis software (Lispix; National Institute of Standards and Technology, Gaithers-burg, MD, USA) [18]. Nowadays, images are processed by the Clinician Report Generator Software (OcuSciences, Inc., Ann Arbor, MI, USA) to display the results [70].

The results are displayed as a histogram of pixel intensities on a 256-U scale [15]. Two primary metrics are recorded. The FPF intensity is the average fluorescence signal within a 5.5 mm diameter area centered on the macula or ONH. FPF heterogeneity is a measure of signal variation or standard deviation in the same region [19]. Heterogeneity has been used since the early second-generation devices, with Field et al. (2012) previously using the width of contrast transitions to quantify the spatial scale over which intensity differences occur [18,25,26,27,28].

### 4.4. FPF in Healthy Eyes

**Figure 3:**
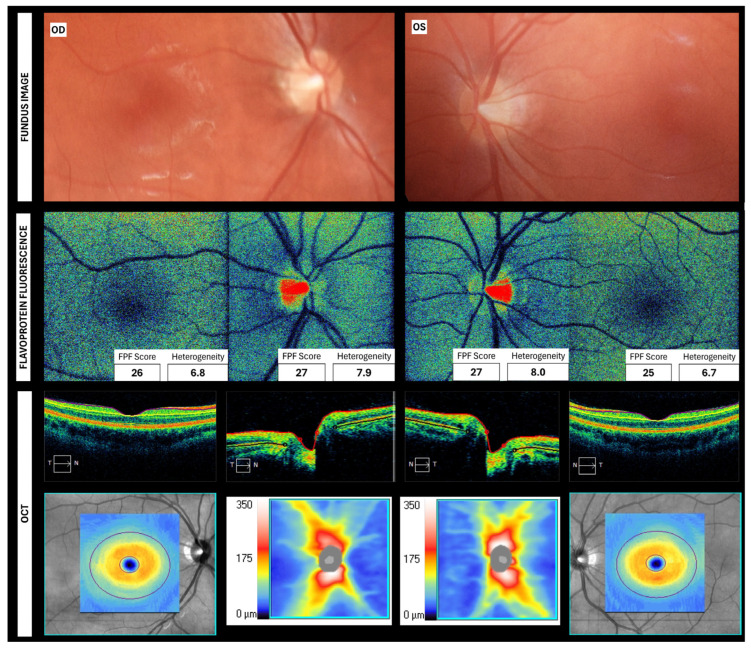
Flavoprotein fluorescence map from a healthy 25-year-old woman, along with further imaging from the same area. The flavoprotein fluorescence profile shows intensity (in grey-scale units) and heterogeneity values for each region in the right inferior corner. Images were taken in our laboratory. OD: Right eye, OS: Left eye, GCL: Ganglion cell layer, IPL: Inner plexiform layer, RNFL: Retinal nerve fiber layer.

Studies have established part of the FPF profile in healthy individuals (See Table 4 and Supplementary Table S4), who consistently show low-intensity, stable, homogeneous, and uniform green-blue fluorescence signals across the macula and optic nerve (See Figure 3). Macular signal tends to have a more diffuse and homogeneous distribution; it is also described to be evenly illuminated without hotspots [12,14,15,16,19,22,23,27]. The green illumination decreases as it moves toward the foveal pit [14]. Ultrastructurally, thinning of several retinal layers has been reported to be associated with higher FPF values, a correlation that is highly age-determined [15].

At the ONH, a slightly higher and more variable FPF signal has been observed, attributed to elevated mitochondrial density in RGC axons [12,15]. Additionally, the temporal region exhibits higher FPF intensity than the other zones, probably due to a higher mitochondrial concentration at the papillomacular bundle [12]. Around the ONH rim, the signal is described as symmetric and uniform [21].

The mitochondrial structure and metabolism in the eye fundus exhibit particular features and undergo dynamic modifications influenced by various factors. The more commonly reported include age, sex, race, smoking status, and intraocular pressure (IOP); several other factors are under study, such as triglycerides, estrogen, and dietary intake [13].

Age has been reported as a key modifier of FPF, as it influences mitochondrial dynamics. In healthy retinas, almost all energy generated at mitochondria is destined for the homeostasis of transmembrane ion gradients. As the lifetime progresses, electron transport chain activity decreases, and reactive oxygen species (ROS) production (e.g., reactive oxygen intermediates, bioreactive lipids, lipid peroxides, and nitric oxide) slowly increases due to light, oxygen, and high turnover of lipid-rich cellular membranes. Mitochondrial DNA, lipids, and proteins are the main structures affected by these oxidative changes; ultimately, they accumulate and alter mitochondrial integrity. This occurs in parallel with a feedback cycle of inflammatory induction that produces more ROS. Despite the compensatory activity of intrinsic antioxidants and anti-inflammatory molecules, progression continues, with older patients showing higher retinal oxidative rates [24,29].

In several cross-sectional studies [15,16,18,21,23,27,28], older age has been reported to be significantly positively correlated with higher FPF intensity in healthy populations and in diseased eyes, thereby confirming the previously described changes. Nevertheless, heterogeneous findings were observed across age groups, with some age groups not significantly different from others [28]. In studies using the first-generation device, the lipofuscin spectrum signal was thought to affect overall results, as it accumulates with aging, and the device’s filters were less effective than those in later designs [27,28].

Males have been shown to have lower oxidative mitochondrial activity than females. This is apparently due to higher ROS production, enhanced apoptosis, and increased iron deposits and metabolism in females [13,23]. Apparently, African American populations are also at less risk of mitochondrial oxidative damage [13]. Long-term retinal ROS exposure from tobacco seems to affect mitochondrial function, which is also related to AMD progression [13]. However, there is no consensus, as multivariate analysis indicates that only age predicts FPF intensity [23]. Other clinical features, such as IOP, eye laterality, and history of posterior vitreous detachment, have not been correlated with FPF metrics [23].

The phakic status has been widely studied and found to be significantly correlated [13]; some studies have even found that the pre-cataract and cataract stages also modify it. Other variables include the presence of an epiretinal membrane and the thickness of the retinal layers themselves, as a thicker layer does not necessarily indicate a higher mitochondrial number or activity.

### 4.5. Potential Signal Confounders

Because some eye structures and components interfere with the FPF signal, current studies adjust imaging settings and use improved algorithms to isolate the signal from the target structure. However, some percentage of these signals may persist, potentially leading to misreadings [12].

The crystalline lens contains tryptophan (295 nm excitation, 329 nm emission) and non-tryptophan (368 nm excitation, 437–523 nm emission) fluorophores; these and other components exhibit a fluorescence spectrum of 430–480 nm that is closer to the flavoproteins, especially in individuals with advanced cataracts, where more lipid fluorophores are also present [14,23]. A similar effect is observed in pseudophakic patients, who exhibit a negative correlation with FPF signals in several reports; it is thought that the blocking filters in intraocular lenses could also interfere with both the device’s output and input signals [13,14,23].

Corneal components are also considered to cause some interference. Flavoproteins present at the epithelium and endothelium, which contain NADH and NADPH, can get excited at a peak near 300–360 nm. Additionally, glycosylated collagen exhibits excitation and emission peaks at 370 and 440 nm, respectively [23].

Lipofuscin is another significant potential confounder; this fluorophore is bright, with a peak excitation at 510 nm, producing green light at 620–630 nm that may overlap with the FPF signal [14]. It is widely found in the RPE and drusen and accumulates in several diseases, such as AMD [23]. The principal fluorescent component found in building up lipofuscin in RPE is N-retinylidene-N-retinylethanolamine [25]. Some studies have reported its signal being captured. Proposing that it may be a machine defect or that the cells are passing through different pathological processes at the same time [6,25,26]. Others, however, report that lipofuscin received less intervention due to disease-specificities and study methodology aimed at avoiding it [25,26].

Melanosomes in the retinal pigment have also been thought to alter the final FPF signal; however, some model designs suggest these are indeed non-fluorescent [13,14]. Other minor fluorophores described are vitamin A, NADH, NADPH, melanin, Bruch’s membrane collagen and elastin, and advanced glycation end products [23]. However, these fluorophores are less linked to FPF because their fluorescence intensity does not increase with increasing oxidative stress [23].

### 4.6. FPF in Eye Diseases

Across the published literature, FPF imaging was applied to a wide spectrum of ocular diseases, including optic disc drusen (ODD) [12], glaucoma suspects (GS) [14,16], POAG [14,16], diabetes mellitus (DM) [23,27,28], non-proliferative (NPDR) [19,23] and proliferative (PDR) [19,23] diabetic retinopathy with or without diabetic macular edema, AMD [13,19,25,28] (early [13], intermediate [13], geographic atrophy [13,25], and neovascular forms [13,19]), central serous retinopathy (CSR) [19,26,28], retinal vein occlusion (RVO) [19], inherited retinal degenerations such as Stargardt disease (STGD) [22] and RP [22,28], papilledema [18], and mitochondrial disorders including MELAS [22] and MT-ATP6-associated disease [22] (See Table 2, Table 5 and Table 6, and Supplementary Table S5 for detailed information on each disease for Section 4.6.1 and Section 4.6.2).

The studies consistently applied heterogeneous yet rigorous criteria for defining control groups, thereby minimizing confounding from systemic or ocular conditions known to influence mitochondrial metabolism or autofluorescence. The matching process in those using control groups was mostly based on age [12,13,14,16,18,19,21,22,23,25,26,27,28].

Conditions such as POAG [14,16] and diabetic retinopathy (DR) [19,23,27,28] used validated and reproducible diagnostic frameworks (e.g., cpRNFL thresholds and ETDRS criteria), improving internal consistency. Other disorders—CSR [19,26,28], ODD [12], and mitochondrial disorders [22]—were diagnosed primarily by specialist interpretation and multimodal imaging without universally accepted grading systems. Genetic confirmation in IRD studies significantly strengthens diagnostic accuracy but is not feasible across all disease types. Overall, diagnostic variability is a key driver of heterogeneity in FPF research studies.

Inclusion and exclusion criteria similarly lacked uniformity, with some studies enforcing strict control of media clarity, comorbid retinal pathology [12,13,14,15,16,19,20,21,22,23,24,25], recent surgery [13,14,15,16,19,20,21,22,23,24,25], or systemic inflammatory disease [13,14,15,16,19,20,21,22,23,24,25], while others [18,26,28] provided minimal detail.

Severity classification was present only in diseases with established grading systems—POAG [14,21], DR [19,23,24,25], RVO [19], and AMD [13,19,25]—and was absent in others such as CSR [26,28], ODD [12], IRD [22,28], and mitochondrial disorders [22]. This creates uneven granularity in disease characterization, limiting the ability to evaluate dose–response relationships between disease severity and FPF signal.

Not all studies used the third-generation FPF device, which may explain differences in detected fluorescence intensity. Acquisition parameters were not uniform, including reported pupillary dilation status [12,13,14,16,18,19,21,22,23,24,25,26,27,28] and the number of frames acquired per eye [13,14,16,20,21,22,23,24,25,26,27,28]. Additionally, reporting of quality-control criteria was inconsistent [12,13,14,16,19,20,21,22,23,24,27], limiting reproducibility across studies.

Imaging outcome definitions were also heterogeneous. Intensity was most commonly defined as the mean grayscale value derived from pixel-intensity histograms within a predefined region of interest [13,14,15,16,18,19,22,23,25,26,27,28]. However, some studies [12,21] evaluating the optic nerve head used protocol-specific adjustments to define the region of interest and the intensity outcome. Heterogeneity metrics varied across studies, including histogram average curve width [18,19,26,27,28], spatial dispersion-based measures [6,13,21,22,23], stress index [15], standard deviation of intensity [14,25], and sectorial analysis [12]; this precludes a direct quantitative comparison. Post-processing pipelines varied, with analyses performed using proprietary imaging software, depending on device generation [13,14,16,19,20,22,24,26] or external platforms [6,12,15,18,27,28], highlighting the absence of standardized analytic workflows. FPF imaging was frequently integrated with optical coherence tomography [6,12,14,15,16,19,20,21,22,23,24], fundus autofluorescence [6,12,24], and visual field testing [12,14,16,20,21] to enable multimodal correlation of metabolic, structural, and functional parameters.

Only one author [19] evaluated FPF using the Area Under the Receiver Operating Characteristic curve; they reported that the technique was sufficiently effective (FPF intensity = 0.989 and FPF heterogeneity = 0.901) at discriminating between diseased and healthy eyes in their cohort.

#### 4.6.1. Retinal Diseases

##### Diabetic Retinopathy

The activation of the polyol pathway, the formation of advanced glycation end products, and the activation of protein kinase C signaling are key hallmarks of hyperglycemia-induced DR. These pathways drive excessive ROS production, leading to mitochondrial fragmentation, impaired oxidative phosphorylation, cytochrome c release, and activation of apoptotic pathways. Ultimately, this process contributes to retinal neurovascular damage and disease progression [23].

These changes are expected to manifest as an increase in the retinal FPF signal. In this regard, some studies have sought to characterize the retinal metabolic profile in DM. Elner et al. (2008 [28]) first observed this in a small subset of DM eyes (14) compared with controls, with elevated FPF signals even in the absence of visible retinopathy. Their DR group also had higher FPF intensity and ACW than the non-DR group. These findings suggested that mitochondrial stress occurs early in DM, before retinal damage or cell death [28].

Following that statement, other authors concurred that DM, with or without DR, exhibited higher FPF signals than healthy eyes [19,27], particularly in DR [23]. Field et al. (2008 [27]) and Chen et al. (2021 [23]) also identified a pattern in which this rise intensified with age. They also showed that FPF elevations were independent of acute glycemic fluctuations, supporting the interpretation that FPF reflects chronic, cumulative metabolic stress and may be a stronger marker than HbA1c [27].

Ahsanuddin et al. (2023) linked the high prevalence of macular edema in their DR group to increased FPF, driven by blood–retinal barrier breakdown, microangiopathy, ischemia, and vascular leakage, which together lead to metabolic stress similar to that in advanced NPDR and PDR [19]. Visual acuity was also found to be worse in DM patients with higher FPF intensity [19] and greater heterogeneity [19,23]. Insulin use was reported to be positively correlated with FPF heterogeneity, and African American heritage was associated with a protective effect. Variables that do not appear to be associated with FPF in DM were gender, smoking status, and clinically significant macular edema [19].

The utility of FPF as a marker of treatment effectiveness was tested by Romo et al. (2018). In a small case series, they compared changes in FPF after intravitreal anti-VEGF therapy (Bevacizumab), concluding that it detects functional improvement after 7–28 days, even when BCVA and OCT changes were non-significant, which reaffirms the previous thought that FPF change precedes OCT sensitivity in predicting visual response [24]. From there, future studies should take a longitudinal approach to clarify and confirm the previously mentioned findings.

However, some discrepancies and limitations have been identified among the authors. Chen et al. showed that FPF was independently associated with DR, while Ahsanuddin et al. stated no difference between NPDR and PDR in terms of FPF signals. Also, not all the authors evaluated both intensity and heterogeneity [24], and Field et al. used the first-generation device, making it difficult to compare with newer models [27]. Finally, they conducted various statistical analyses, further complicating homogenization [19,23,24,27].

##### Retinal Vein Occlusion

This condition is characterized by acute venous outflow obstruction, retinal ischemia, and oxidative injury. Mitochondrial complex I is reported to be predominantly damaged at the retinal level, secondary to ischemia [44]. This highly elevated metabolic stress was observed in vivo by Ahsanuddin et al. (2023 [19]), who reported significantly higher macular FPF intensity than in healthy controls. As with the problem encountered in DM, FPF was unable to differentiate between CRVO and BRVO [19]. One potential explanation is that RVO might cause a more uniform metabolic insult, driven by acute ischemia, rather than a varied, cell-specific mitochondrial injury. Patients with worse visual acuity had higher FPF signals [19].

##### Central Serous Chorioretinopathy

In patients with CSR, there is little literature about specific mitochondrial alterations happening in the retina. Well-known pathophysiological events include choroidal hyperpermeability or RPE dysfunction caused by mineralocorticoid receptor activation, vortex vein compression, and dysregulation of complement or adrenergic pathways. Nevertheless, oxidative stress is a key mechanism of damage to RPE and choroidal endothelial cells and is intrinsically linked to mitochondrial damage. As a result, elevated FPF signals are expected [72]. Higher macular FPF intensities in affected eyes than in controls have been reported in some studies [19,26,28], and these intensities were also correlated with worse vision in Ahsanuddin’s cohort [19].

Field et al. (2009 [26]) proposed that, in the presence of calcium, ceramide is produced and, in turn, permeabilizes the mitochondrial outer membrane, leading to apoptosis. This lowers the membrane potential, causing the oxidation of flavoproteins [26]. As in previous studies, chronically inactive CSR patients do not show differences in macular FPF intensity or heterogeneity compared with healthy people. Intensities in active CSR and chronic CSR were also not statistically different. This suggested that mitochondrial metabolism tends to normalize despite structural adaptive cellular changes derived from disease [19].

##### Age-Related Macular Degeneration

Mitochondrial damage in AMD is characterized by decreased oxidative phosphorylation, increased ROS generation, and accumulation of mitochondrial DNA mutations. Mitochondrial structure is altered; fusion–fission balance is impaired; mitophagy is reduced; and they accumulate in photoreceptors and RPE cells. Studies have reported a decline in the number of RPE mitochondria and mitochondrial cristae, as well as in total mitochondrial surface area. Ultimately, they disintegrate [25,73,74]. The age-related maculopathy susceptibility 2 gene has also been related to mitochondrial damage [75]. Oxidative stress induces changes in ΔΨm, thereby oxidizing flavoproteins and increasing fluorescence relative to healthy maculae across AMD stages [13,19,25,28].

In intermediate dry AMD cases, Susan Elner et al. (2008 [28]) and Field et al. (2012 [25]) were the first to suggest FPF as a means to characterize metabolic impairment that occurs before structural damage, with the latter showing a regional, non-uniform distribution of signals. Early AMD eyes showed FPF intensities similar to those of healthy eyes, whereas the heterogeneous fluorescence distribution in the cases differed markedly [13].

Eyes with GA are reported to have the highest intensity and heterogeneity in AMD; however, no formal comparison has been conducted. Their localized hyperfluorescent areas are more prominent at the periphery and within the GA zones, indicating that even the remaining mitochondria that persist are highly stressed [25]. Neovascular AMD was reported to have a macular FPF intensity more than double that of healthy controls [19].

Signal heterogeneity may be better suited for tracking disease activity in AMD, given the disease’s natural history, as it may reflect RPE damage rather than retinal alterations. Several areas with a higher FAF signal corresponded to those with higher FPF [13]. Since no correlation between retinal thickness and FPF was encountered, the idea that metabolic imaging precedes the structural findings is supported [13].

Visual acuity was reported to be low, both qualitatively and quantitatively, with higher FPF signals [19,28]. Age was a positive predictor of intensity and heterogeneity, along with smoking status. Conversely, the black race was protective, and pseudophakia reduced the intensity but increased the heterogeneity detected by the device [13].

##### Stargardt Disease

In STGD, the accumulation of all-trans-retinal due to impaired ABCA4 provokes gasdermin E activation, which, conversely, damages photoreceptor mitochondria. Animal models showed that these cells accumulate ROS, iron, and lipid peroxides [76]. All this occurs alongside the well-known oxidative stress driven by lipofuscin accumulation and toxic retinoid byproducts in the RPE [77].

**Figure 4:**
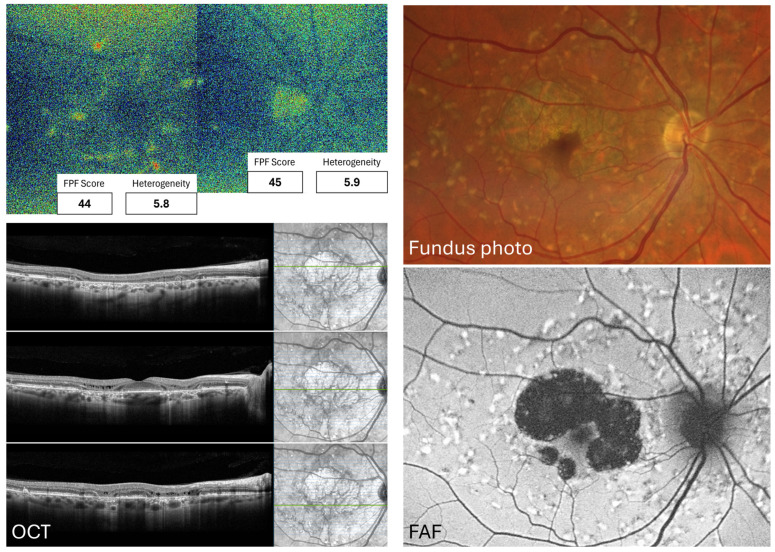
Representative images from the right macula and ONH from a 57-year-old female with Stargardt disease, displaying a macular intensity of 44 gsu and a heterogeneity of 5.8, and an ONH intensity of 45 gsu and a heterogeneity of 5.9. She carries an ABCA4, heterozygous pathogenic mutation c.768G>T (p.Val256=) and ABCA4, heterozygous risk factor mutation c.5603 A>T (p.Asn1868Ile). Images were taken in our laboratory. ONH: Optic nerve head, gsu: Grey-scale units. FPF: Flavoprotein fluorescence, OCT: Optical coherence tomography, FAF: Fundus autofluorescence. ABCA4: ATP Binding Cassette Subfamily A Member 4, G: Guanine, T: Thymine, A: Adenine, Asn: Asparagine, Ile: Isoleucine.

The higher macular FPF intensity and heterogeneity in ABCA4 gene-related STGD patients than in age-matched controls were first reported by Russell et al. (2022 [22]) and subsequently corroborated by Merle et al. (2025 [6]); see Figure 4 for an example from one of our patients. Nevertheless, given that the lipofuscin signal overlaps with the FPF spectrum [14], this disease presents characteristics that require modifications in the study approach and methodologies. The first author stratified SD lesions into three groups based on FAF appearance, finding that FPF signal overlaps areas of abnormal FAF but that independent, higher FPF signals were also present [22].

Merle et al. made a step forward by correlating FPF with both OCT and FAF patterns. They found that the FPF signal arises more commonly in photoreceptor outer segments, but a significant portion also originates from lipofuscin. Additionally, other layers, such as the ganglion cell layer, contributed to the fluorescence signal, and, as before, a considerable portion of that signal originated from lipofuscin [6]. The various lesions observed in these patients demonstrated how dynamic changes affect the FPF signal. Healthy retinas exerted normal FPF, FAF, and OCT signals. As damage progresses, IZ layer loss shows a slight FPF elevation that peaks with the EZ line impairment. Then, when retinal atrophy stabilizes, the FPF signal drops [6].

##### Rod–Cone Dystrophies

Early metabolic oxidative stress, structural alterations, and progressive dysfunction precede photoreceptor death in rod–cone dystrophies. There is lower oxygen consumption, aberrant calcium signaling, and mitochondrial swelling. Autophagy is also impaired, allowing damaged mitochondria to remain active in cells [78,79].

**Figure 5:**
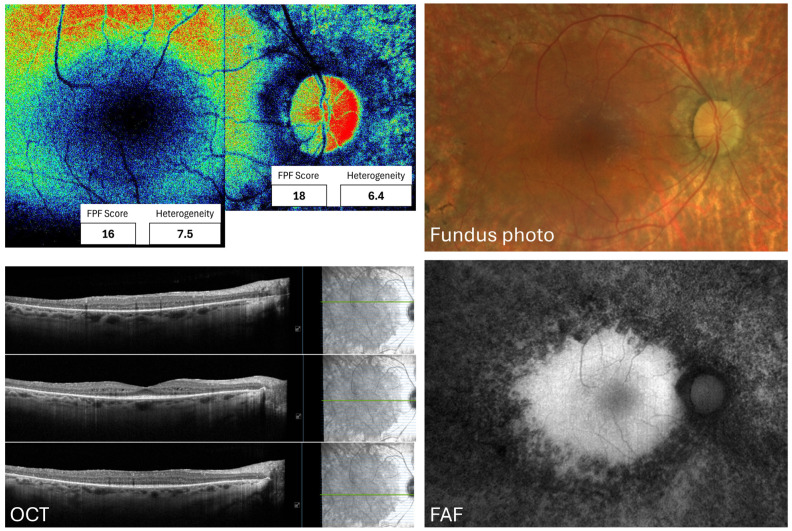
Images from the right eye of a 58-year-old female with retinitis pigmentosa displaying a macular intensity of 16 gsu and a heterogeneity of 3.8, and an ONH intensity of 18 gsu and a heterogeneity of 6.4. She presents with four heterozygous pathogenic mutations, two at USH2A gene (c.10073G>A (p.Cys33587Tyr) and c.2299del (p.Glu767Serfs^21)), one at ADGRV1 gene (c.6901C>T (p.Gln2301)), and one at RPE65 gene (c.271C>T (p.Arg91Trp)). ONH: Optic nerve head, gsu: Grey-scale units, USH: Usher syndrome, FPF: Flavoprotein fluorescence, OCT: Optical coherence tomography, FAF: Fundus autofluorescence, G: Guanine, A: Adenine, Cys: Cysteine, T: Thymine, RPE: Retinal pigment epithelium, Arg: Arginine, Trp: Tryptophan, del: Deletion, Glu: Glutamic acid, Adhesion G protein-coupled receptor V1.

The superior macular FPF intensity, heterogeneity, and ACW in patients compared with controls have been studied in vivo using FPF, yielding new insights into mitochondrial metabolic behavior in these patients [22,28]. In RP retinas, a significant FPF signal occurs at the lesion periphery compared with FAF intensities. Also, FPF signals are found in both hyper- and hypo-fluorescent FAF regions, and they seem to arise from regions with outer retinal degeneration. However, FPF signals did not significantly predict visual acuity [22]. Check Figure 5 for an example taken in our laboratory.

##### Other Inherited Retinal Diseases

Other conditions, such as BBS, MELAS, and MT-ATP6, have also been studied using FPF by Russell and collaborators, but with a small number of patients. Macular intensity was only non-statistically significantly higher in MELAS. Macular heterogeneity was only superior in BBS retinas. Finally, they found contrast between lesion intensities in MT-ATP6 that was not previously noted on FAF imaging [22].

#### 4.6.2. Optic Nerve Diseases

##### Glaucoma

Glaucomatous eyes exhibit reduced mitochondrial metabolic activity and increased mitochondrial mutations compared with healthy eyes [14]. RGCs present with mitochondrial remodeling and redistribution, accompanied by volume reduction [80]. Animal models have shown that, early in chronic glaucoma, mitochondria tend to increase in concentration but ultimately decrease in number and size [36]. These structural changes occur in parallel with functional changes, such as diminished basal or maximal mitochondrial respiration and reduced spare respiratory capacity [80]. In turn, ATP production decreases by 23%, leading to a deficiency in oxidative phosphorylation. This is accompanied by at least a 30% rise in superoxide and hydrogen peroxide production [42]. Additional alterations include augmented mitochondrial fission and impaired mitophagy, leading to the accumulation of dysfunctional mitochondria containing damaged mtDNA [81,82]. Furthermore, damaged mitochondria activate inflammatory pathways, creating a positive feedback loop of impairment that accelerates RGCs’ neurodegeneration, culminating in their apoptosis [16,83].

The fact that POAG is a silent disease has prompted FPF to be studied as a potential biomarker of early disease, proposing that it could detect pre-apoptotic oxidative stress on RGCs and at the ONH [14]. Nonetheless, both macular and ONH FPF analyses have yielded inconsistent results across studies. At the macula, Geyman et al. (2018 [16]) reported significantly higher FPF intensity in GS eyes compared with controls; nevertheless, there was no significant difference in the POAG group. A further analysis using the relative metric FPF/GCL+IPL showed a highly significant difference between the groups and the controls. They also encountered foci of hyperfluorescence, consistent with the focal progression of the disease [16]. This suggested that metabolic impairment could be detectable before the structural one, and that cellular loss could mask the rise in FPF signals. However, Caro et al. (2025) did not observe any significant difference in macular FPF, whether considered alone or as an index, in both POAG and GS eyes when compared to controls [14].

Previously, Zhou et al. (2022) reported significantly superior FPF metrics in glaucomatous ONH rims, particularly in the temporal region [21]. In contrast, Caro et al. (2025) found no significant differences in ONH and macular fluorescence parameters between GS patients, those with POAG, and controls, even after normalization by RNFL thickness, including the temporal region [14].

No association between FPF and IOP in the macula [14,16] or at the ONH [21] has been reported, likely due to prior use of ocular antihypertensive therapy, which may exert neuroprotective effects on the optic nerve [21].

At the structural and functional levels, no clear consensus has emerged. Zhou et al. found significant negative correlations between circumpapillary RNFL and FPF globally and by sector, except temporally [21]. Conversely, both Caro et al. and Geyman et al. encountered no associations between FPF and macular thickness, global or temporal RNFL thickness, or visual field parameters (MD, PSD), nor with pseudophakia status. Age was the only consistently positive correlate of higher ONH FPF intensities [14,16,21].

Sun and colleagues used FPF to track treatment effectiveness. After 1 month of pre–Balance Goggles System application, FPF intensity at the optic nerve rim lowered significantly. When analyzing the optic disc as a whole, the difference was not statistically significant [20]. This heightened concern about the sensitivity of the measurement area, including other non-objective mitochondria, could bias the results.

##### Papilledema

The edema present at the ON papilla has specific effects on RGCs and, consequently, on mitochondrial dynamics. The RGCs’ axons experience axoplasmic transport stasis due to elevated intracranial pressure or microvascular ischemia, creating metabolic stress. Mitochondria accumulate in the prelaminar zones of swollen axons, and energy metabolism becomes impaired [84]. At the macula, the GCL-IPL thins progressively from the very early stages, along with changes in vascular perfusion. This is closely related to impaired mitochondrial function [85].

**Figure 6:**
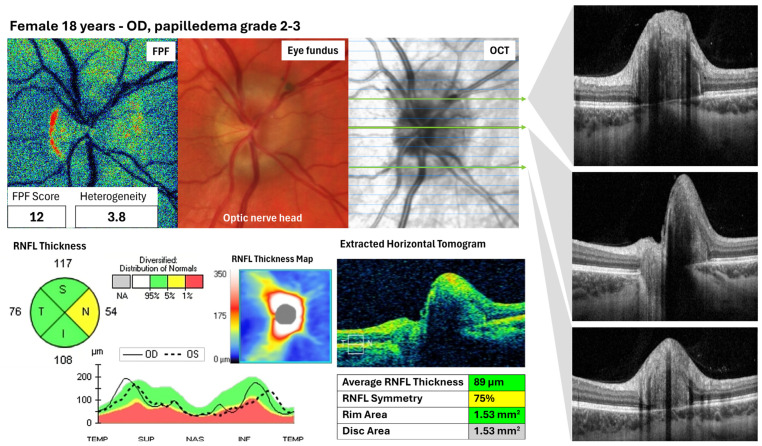
Images from the OD ONH from an 18-year-old female with grade 2–3 papilledema, displaying an intensity of 12 gsu and a heterogeneity of 3.8. Images were taken in our laboratory. ONH: Optic nerve head, gsu: Gray-scale units, FPF: Flavoprotein fluorescence, OCT: Optical coherence tomography, FAF: Fundus autofluorescence, RNFL: Retinal nerve fiber layer, S or SUP: Superior quadrant, NAS or N: Nasal quadrant, I or INF: Inferior quadrant, T or TEMP: Temporal quadrant.

To our knowledge, only Victor Elner et al. (2008 [18]) in their first report on FPF in the human eye fundus studied papilledema. Using the prototype, they found that the maculae of 12 untreated eyes had higher foveal intensity than those of healthy age-matched controls. It is important to note that they did not assess the ONH. Additionally, when comparing the same patient’s eyes, the more affected eye had significantly higher intensity than the contralateral eye, a finding not observed in the control group [18]. In Figure 6, we present a case of papilledema grade 2–3 in which the FPF intensity was lower than the reported normal range for controls.

##### Optic Disc Drusen

In this condition, a combination of hypoxia-driven ATP deficiency and mineral deposit overload could trigger mitochondrial dysfunction. This may initiate or accelerate ectopic mineralization [86]. Afterwards, mitochondria are apparently extruded from cells when exons die due to calcified bodies-induced damage, serving as a source of further calcification [87]. Electron-dense deposits have been found in ODD mitochondria, suggesting that their calcification and dysfunction are among the earliest changes. Calcium and phosphate-rich matrix vesicles from those organelles would be released into the extracellular environment [88].

In this context, a recent study by Pujari et al. (2026 [12]) found that ONHs from patients with superficial or buried ODD showed higher FPF intensity than those from controls; macular intensities did not differ between groups. They showed that hyperintense areas inside the ONH correlated with FAF hyperautofluorescence and with the typical lesions observed on EDI-OCT. The nasal sector had the highest FPF intensity within the ONH. A negative correlation was also observed between FPF intensity and RNFL or GCC thickness, suggesting that FPF intensity increases as those layers thin. Interestingly, a non-linear negative correlation was found with the VF MD [12].

However, the study did not fully distinguish FPF from drusen-related autofluorescence. The observed signal likely represents a composite of intrinsic drusen fluorescence and mitochondrial metabolic stress associated with axonal injury. Although methodological approaches including optical filtering, region-of-interest segmentation, and multimodal imaging were used to reduce confounding, signal overlap persisted. Consequently, elevated FPF in ODD should be interpreted as reflecting both structural and metabolic contributions, limiting specificity as a purely functional biomarker.

## 5. Discussion

### 5.1. Strengths and Limitations of FPF

FPF imaging offers a promising approach to assess retinal metabolism and provides a novel strategy for understanding and potentially guiding the treatment of ocular diseases. However, several limitations have been identified across studies.

While FPF seems to be a good discriminator between disease and healthy states, the ability to distinguish among different diseases (e.g., PDR versus NPDR, AMD versus RVO) [19,23,27,28] remain limited, highlighting the need to integrate FPF with complementary diagnostic modalities to establish a more comprehensive accurate disease profile. As described above, each retinal layer has a different mitochondrial concentration per cell, and current FPF imaging cannot localize the signal origin from each layer [14]. Additionally, FPF imaging cannot reliably differentiate temporal pathophysiological processes. For example, in CSR, the signal could reflect primary metabolic stress on retinal cells, serous detachment, or both [26].

Some diseases, such as AMD, are associated with fewer mitochondria in the retina. In this context, some studies opted to correct the fluorescence metrics relative to healthy controls to better maintain an analysis of mitochondrial function rather than merely quantify structural differences [25].

Despite the advances made by Kim et al. in characterizing FPF profiles in healthy populations, discrepancies remain between their reported correlations of FPF with other imaging modalities, such as OCT and FAF, and those observed in other studies. These differences may be partly explained by methodological heterogeneity, variations in inclusion and exclusion criteria, and, importantly, differences in device design and configuration. Although most studies used third-generation FPF devices, the specific imaging systems were not identical across laboratories or clinical centers. This highlights the need for a standardized protocol for conducting projects with this technology.

Current studies rely mostly on cross-sectional analyses of patients rather than time-based follow-ups. Some studies have employed multivariate analyses of variables related to FPF but causal relationships remain unclear [12,13,14]. Additionally, data on early or untreated disease is limited restricting the characterization of disease-specific metabolic patterns, and the inclusion of mostly treated patients may further confound FPF measurements [14].

The lack of standardization of FPF-derived measures, indices, segmentation strategies, and analyzed fundus regions limits the replicability and comparability of findings across studies, even within the same disease category. Most studies adapted their analytical approach according to the disease stage under investigation, the structural alterations present in the cohort, and the software tools available for each device version. Based on the available literature, mean FPF intensity and heterogeneity appear to be the most consistently reported and practically comparable parameters across studies. Further refinement of segmentation and focus-area measurement is needed to reduce bias and improve reproducibility.

Another limitation relates to the spatial distribution of lesions and their correlation with FPF and functional measures. High FPF intensity may be present, but if the foveal photoreceptors remain functional, visual acuity is preserved [13]. On the other hand, in regions with degenerated retinal layers, the detected signal could come from the remaining functional layer, which is also subject to oxidative stress, or from deeper structures such as the choroid [25]. A degenerated or absent retinal layer could also alter incoming and outgoing signals, further complicating the interpretation of FPF measurements.

Recent studies focusing on the ONH track only the papillary area rather than the peripapillary zones, as in previous studies. This improves metric accuracy and facilitates the study of organ-specific diseases such as papilledema and ODD [12]. However, the heterogeneity in the analysis (e.g., optic rim vs. optic disc) limit comparability and generalizability across studies.

### 5.2. Scoping Review Process Limitations

In this scoping review, not all studies disclosed the raw data underlying several reported results, and time constraints prevented us from contacting all corresponding authors. The absence of longitudinal studies and the methodological heterogeneity across the included studies precluded a more in-depth systematic analysis. In addition, the time span between the earliest and most recent studies, together with subsequent technological improvements in the device, heterogeneous image acquisition, and structural focus measurement methods, limited direct comparability of findings and precluded data harmonization.

### 5.3. Future Perspectives

Future studies should incorporate longitudinal designs to better characterize disease progression and perform more robust correlational analyses between FPF signals and complementary functional and structural imaging modalities. For instance, although the BCVA and FPF metrics are reported to be significantly correlated, the cross-sectional nature of most analyses cannot determine whether these changes occur concurrently [13].

After establishing healthy and disease-specific FPF profiles, integration into clinical practice may be considered. Potential benefits of this approach include improved staging of disease severity, improved patient selection for studies, and a better understanding of the relationship between severity of the disease and vision function [12]. FPF may also serve as a tool to track treatment efficacy, such as evaluating neuroprotective strategies in POAG [21]. However, individualized assessment of imaging findings would still be needed given the complexity and disease-specific variability of FPF signals [13].

## 6. Conclusions

FPF imaging of the ocular fundus is a novel technique that provides new insights into retinal and optic nerve diseases as well as the underlying pathophysiology and natural history of both affected and healthy eyes. However, substantial heterogeneity exists across studies in both patient cohorts and methodologies, including variability among control groups and within the same disease categories. Larger, more standardized populations are needed to establish reliable and generalizable FPF profiles.

Further technological refinement of imaging systems, along with longitudinal analysis of large cohorts of patients and healthy individuals are needed. A comprehensive integration of FPF with complementary diagnostic modalities will be critical to improve interpretation and clinical applicability. Normal structural findings on OCT or FAF, accompanied by FPF anomalies, could help the future ophthalmologist adopt an early-disease approach. FPF intensity and heterogeneity metrics appear informative, depending on disease features. Retinal metabolic distress does not always precede structural damage given mitochondria resilience may vary across conditions and disease stage.

## Figures and Tables

**Table 1:** Summary of total demographics.

	Total Number
**Subjects (total)**	**1339**
*Controls*	639
*Patients*	700
**Eyes (total)**	**1914**
*Controls*	898
*Patients*	1016

**Table 2:** Summary of selected studies for analysis and their objectives.

PMID	Author	Setting	Design	Recruitment Period	Device Used (Generation)	Objective of the FPF Study	Structure	Control Group
41260483	Pujari et al. 2026 [12]	Byers Eye Institute at Stanford University Medical Center, USA	Prospective cross-sectional	May 2021–October 2024	OcuMet Beacon (3rd)	- FPF in ODD * and correlation with VF and OCT	ONH	Yes
40767444	Merle et al. 2025 [6]	University Eye Clinic, Tübingen, Germany	Cross-sectional prospective	June 2023–January 2024	OcuMet Beacon (3rd)	- FPF qualitatively and quantitatively in ABCA4-Stargardt disease *, and correlation with FAF and OCT	Macula	No
40631437	Kim et al. 2025 [15]	University of Miami Miller School of Medicine, USA	Observational cross-sectional	Not mentioned	OcuMet Beacon (3rd)	- FPF and its relations with OCT intraretinal thicknesses in healthy adults *	Macula and ONH	No
39808681	Caro et al. 2025 [14]	University of Washington Medicine Eye Institute, USA	Cross-sectional observational	April 2023–December 2023	OcuMet Beacon (3rd)	- FPF in normal, GS, and POAG eyes. Correlation with VF and OCT	Macula and ONH	Yes
36626211	Muste et al. 2023 [13]	Cleveland Clinic Foundation, USA	Observational cross-sectional	August 2018–December 2021	OcuMet Beacon (3rd)	- FPF in AMD stages *	Macula	Yes
38983095	Ahsanuddin et al. 2023 [19]	New York Eye and Ear Infirmary of Mount Sinai, USA	Observational cross-sectional	October 2021–August 2022	OcuMet Beacon (3rd)	- FPF in RVO *, DR *, exudative AMD *, CSR *, and healthy eyes *. Assess device sensitivity and specificity in distinguishing controls from diseased subjects. Association of FPF and BCVA in different disease states	Macula	Yes
35696700	Sun et al. (2022) [20]	Stanford University, USA	Prospective comparative case-series	July 2019–March 2022	OcuMet Beacon (3rd)	- IOP-lowering effects of BGS in POAG *, using FPF as a marker	ONH	No
34968754	Zhou et al. (2022) [21]	Einhorn Clinical Research Center of the New York Eye and Ear Infirmary of Mount Sinai, USA	Retrospective cross-sectional	November 2015–October 2016	OcuMet Beacon (3rd)	- FPF in POAG *	ONH	Yes
36384402	Russell et al. (2022) [22]	Cleveland Clinic Cole Eye Institute, USA	Observational cross-sectional	January 2021–December 2021	OcuMet Beacon (3rd)	- FPF in IRDs: Rod Cone dystrophies *, BBS *, MELAS, Stargardt *, and MT-ATP6 *	Macula	Yes
32709959	Chen et al. (2021) [23]	Cleveland Clinic, USA	Observational cross-sectional	August 2018–January 2020	OcuMet Beacon (3rd)	- FPF in DR *	Macula	Yes
29750714	Geyman et al. (2018) [16]	New York Eye and Ear Infirmary of Mount Sinai, USA	Observational cross-sectional	November 2015–October 2016	Scanning laser ophthalmoscope (2nd)	- FPF in GS * and POAG, relative to retinal ganglion cell structure *	Macula	Yes
30159113	Romo et al. (2018) [24]	New York Eye and Ear Infirmary of Mount Sinai, USA	Prospective observational case series	Not mentioned	Scanning laser ophthalmoscope (2nd)	- FPF to monitor the therapeutic effect of anti-VEGF in CSDME *, in addition to fundus photography, BCVA, and OCT	Macula	No
22822904	Field et al. (2012) [25]	University of Michigan W. K. Kellogg Eye Center, USA	Observational cross-sectional	February–June 2010	Scanning laser ophthalmoscope (2nd)	- FPF in AMD * stages	Macula	Yes
19491721	Field et al. (2009) [26]	University of Michigan, USA	Cross-sectional	Not mentioned	Modified Zeiss F4 fundus camera (1st)	- FPF in unilateral CSR *	Macula	Yes
18625939	Field et al. (2008) [27]	University of Michigan, USA	Cross-sectional	June–September 2007	Modified Zeiss F4 fundus camera (1st)	- FPF in DM * with and without DR *	Macula	Yes
18268219	Elner et al. (2008) [28]	University of Michigan, USA	Observational cross-sectional	Not mentioned	Modified Zeiss F4 fundus camera (1st)	- FPF in DM *, AMD *, CSR *, RP *. Assess inter-eye macular signal asymmetry to detect early dysfunction	Macula	Yes
19277237	Elner et al. (2008) [18]	University of Michigan, USA	Observational cross-sectional	Not mentioned	Modified Zeiss F4 fundus camera (1st)	- FPF in papilledema *, assess signal asymmetry as a marker	Macula	Yes

PMID: PubMed Identifier, FPF: Flavoprotein fluorescence, ABCA4: ATP Binding Cassette Subfamily A Member 4, FAF: Fundus autofluorescence, OCT: Optical coherence tomography, ONH: Optic nerve head, GS: Glaucoma suspect, POAG: Primary open-angle glaucoma, ODD: Optic disc drusen, AMD: Age-related macular degeneration, RVO: Retinal vein occlusion, DR: Diabetic retinopathy, CSR: Central serous retinopathy, BCVA: Best corrected visual acuity, IOP: Intraocular pressure, BGS: pre–Balance Goggles System, IRDs: Inherited retinal diseases, BBS: Bardet–Biedl Syndrome, MELAS: Mitochondrial encephalomyopathy, lactic acidosis, and stroke-like episodes; MT-ATP6: Mitochondrial ATP synthase subunit 6 mutation, VEGF: Vascular endothelial growth factor, CSDME: Clinically significant diabetic macular edema, DM: Diabetes mellitus, RP: Retinitis pigmentosa. Diseases or fundus structures in which a significant correlation in FPF metrics was found are in bold, followed by an (*).

**Table 3:** Mitochondria distribution and concentration in the retina [30,31].

Retinal Layer/Sublayer	Mitochondrial Density/ Activity	Key Characteristics
**RNFL**	Moderate–high	Punctate mitochondrial labeling supporting axonal metabolism
**GCL**	High	RGCs’ somas show robust mitochondrial enrichment, even in avascular species, which supports high oxidative demand
**IPL**	High in vascular	Synaptic ribbon activity requires oxidative metabolism; there is strong enzyme activity
**INL—general**	Moderate	Bipolar, horizontal cell bodies contain mitochondria
**INLm**	Highest mitochondrial occupancy in the OPL/INL region	Postsynaptic dendritic zone of bipolar/horizontal cells; corresponds to the inner OPL hyperreflective band on OCT
**OPL—overall**	High	Contains mitochondria in photoreceptor terminals and in dendritic processes
**OPL1a**	High mitochondrial density	Clusters of ovoid mitochondria within cone pedicles and rod spherules
**OPL1b**	Low	Mitochondria-poor zone between photoreceptor terminals and synapses
**OPL2a**	Moderate–high	Dendrites of bipolar/horizontal cells with numerous small-caliber mitochondria
**OPL2b**	Low–moderate	Transition dendritic zone with fewer mitochondria
**ONL**	Negative (no mitochondria)	Photoreceptor somas contain little to no mitochondria
**Photoreceptors:**		
**Inner Segments**	Highest density in the entire retina	Ellipsoid region densely packed with mitochondria; peak oxidative region
**Outer Segments**	None	Outer segments lack mitochondria
**Terminals (synaptic boutons)**	Present	Mitochondria colocalize with synaptophysin in rat/marmoset; missing in rabbit/guinea pig
**Müller cells—vitreal endfeet**	Present	Mitochondria are present in endfeet only when the retinal vasculature is present
**Müller cells—scleral/ELM processes**	Minimal in vascular; prominent in avascular	Avascular retinas show mitochondria only at the ELM, reflecting reliance on an oxygen gradient
**RPE**	High, basolateral clustering	Mitochondria are concentrated at the basal surface adjacent to the choriocapillaris

RGCs: Retinal ganglion cells, RNFL: Retinal nerve fiber layer, GCL: Ganglion cell layer, IPL: Inner plexiform layer, INL: Inner nuclear layer, OPL: Outer plexiform layer, INLm: INL–OPL border, OCT: Optical coherence tomography, ONL: Outer nuclear layer, ELM: External limiting membrane, RPE: Retinal pigment epithelium.

**Table 4:** Reports on FPF metrics in healthy patients.

PMID	Author	Eyes (n)	Age (Mean ± SD)	Device Generation	FPF Intensity (Mean ± SD gsu)		FPF Heterogeneity (Mean ± SD)	
					Macula	ONH	Macula	ONH
40631437	Kim et al. (2025) [15]	147	56.1 ± 21.7	3rd	24.1 ± 12.2	30.6 ± 14.6	SI = 0.96 ± 0.36	SI = 5.34 ± 8.52
39808681	Caro et al. (2025) [14]	25	60.6 ± 17.4	3rd	89.0 ± 27.2	78.8 ± 33.7	18.6 ± 3.8	18.4 ± 3.8
41260483	Pujari et al. (2026) [12]	69	51	3rd	20.78 ± 0.61	4.58 ± 0.21	-	-
40767444	Merle et al. (2025) [6]	25	≈46 ± 14 ^§^	3rd	84 ± 9	-	18.6 ± 2.1	-
38983095	Ahsanuddin et al. (2023) [19]	21	55 ± 9	3rd	30.62 ± 8.03	-	15.62 ± 2.87	-
34968754	Zhou et al. (2022) [21]	36	55 ± 9	3rd	-	28.0 ± 11.7	-	
32709959	Chen et al. (2021) [23]	151	63.5 [53.3, 69.5] ^¥^	3rd	73.0 [56.3–83.8] ^¥^	-	0.51 [0.36–0.67] ^¥^	-
29750714	Geyman et al. (2018) [16]	32	55 ± 7	2nd	327 ± 91	-	-	-
22822904	Field et al. (2012) [25]	3	71–84	2nd	1.0 ± ~0.05 (normalize) ^§^		18.2 ± 2.3 *	
19491721	Field et al. (2009) [26]	6	30–42	1st	≈22 ± 8	-	-	-
18625939	Field et al. (2008) [27]	42	44.7 ± 9.4	1st	28.2 ± 3.1	-	29.1± 2.4 (ACW)	-
18268219	Elner et al. (2008) [28]	38	23–77	1st	15.49 ± 5.26	-	27.21 ± 7.30 (ACW)	-
19277237	Elner et al. (2008) [18]	12	36.5 ± 4.7	1st	≈30.98 ± 8.38 *	-	-	-

SD: Standard deviation, PMID: PubMed Identifier, FPF: Flavoprotein fluorescence, ONH: Optic nerve head, SI: Stress index, gsu: Gray-scale units. * Value calculated from available data in the study results; ^§^ value estimated from available data in the study results; ^¥^ only the IQR range was available.

**Table 5:** Current macular FPF profiles categorized by disease. *p*-values are provided when cases are compared to controls, unless specified otherwise in the table.

Disease	Cases (n)	Eyes (n)	Macular FPF Metrics		Reference
			Intensity (Mean ± SD gsu, *p*-Value)	Heterogeneity (Mean ± SD, *p*-Value)	
POAG	54 (33 mild, 12 moderate, 9 severe)	54	92.7 ± 32.9, *p* = 0.605 *	19.1 ± 5.0, *p* = 0.630 *	Caro et al. 2025 [14]
	20	40	388 ± 118, *p* = 0.240	-	Geyman et al. 2018 [16]
GS/OHT	16	16	93.4 ± 19.5, *p* = 0.551 *	17.4 ± 3.4, *p* = 0.300 *	Caro et al. 2025 [14]
	8	16	**↑**, 437 ± 141, *p* < 0.05	-	Geyman et al. 2018 [16]
AMD	5	6	**↑**, 1.80 ± 0.22 ^§^	29.45 ± 8.25 *	Field et al. 2012 [25]
- Dry AMD:					
Early	≈21	31	*p =* 0.302	**↑**, *p* < 0.001	Muste et al. 2023 [13]
Intermediate	≈63	94	**↑**, *p* < 0.001	**↑**, *p* < 0.001	
	3	3	**↑**, ≈1.6 ± 0.05 (normalized) ^§^, *p* < 0.001	28.3 ± 6.3 *	Field et al. 2012 [25]
	1	2	**↑**, 72.0 ± 27.3 ^§^	**↑**, 61.5 ± 12.5 (ACW) ^§^	Elner et al. 2008 [28]
GA	≈25	38	**↑**, *p* < 0.001	**↑**, *p* < 0.001	Muste et al. 2023 [13]
	2	3	**↑**, ≈2.0 ± 0.3 (normalized) ^§^, *p* = 0.044	30.6 ± 11.3 *	Field et al. 2012 [25]
- Neovascular AMD	≈24	36	**↑**, *p* < 0.001	**↑**, *p* < 0.001	Muste et al. 2023 [13]
	17	17	**↑**, 67.47 ± 17.77, *p* < 0.001	**↑**, 23.12 ± 9.91, *p* < 0.001	Ahsanuddin et al. 2023 [19]
RVO	20	20	**↑**, 53.80 ± 17.97, *p* < 0.001	**↑**, 18.00 ± 4.10, *p* = 0.026	Ahsanuddin et al. 2023 [19]
CRVO	11	11	53.55 ± 21.99, *p* = 0.619 ^φ^	18.27 ± 4.47	
BRVO	9	9	54.11 ± 12.76	17.67 ± 3.84	
DM	117 (101 type 2 DM, 16 type 1 DM)	117	**↑**, 76.0 [67.0–92.0] ^¥^, *p* = 0.002	**↑**, 0.65 [0.48–0.92] ^¥^, *p* < 0.001	Chen et al. 2021 [23]
	21 (15 type 2 DM, 6 type 1 DM)	42	**↑**, 58.4 ± 3.1, *p* < 0.001	**↑**, 55.2 ± 2.4 (ACW) gsu, *p* <0.001	Field et al. 2008 [27]
DM wo DR	63	63	76.0 [62.0–86.0] ^¥^	0.58 [0.43–0.74] ^¥^	Chen et al. 2021 [23]
	9	18	43.5 ± 12.2	44.8 ± 10.5 ACW	Field et al. 2008 [27]
	7 (6 type 2 DM, 1 type DM 1)	14	**↑**, 59.5 ± 10.5, *p* = 0.001	58.7 ± 9.1 (ACW), *p* = 0.08	Elner et al. 2008 [28]
DM, 30–39 yrs	7	14	**↑**, 52.4 ± 6.1, *p* = 0.002	**↑**, 49.0 ± 4.8 (ACW), *p* = 0.001	Field et al. 2008 [27]
DM, 40–49 yrs	7	14	**↑**, 54.2 ± 4.7, *p* = 0.004	**↑**, 52.8 ± 4.4 (ACW), *p* = 0.006	
DM, 50–59 yrs	7	14	**↑**, 68.9 ± 5.7, *p* = 0.001	**↑**, 64.1 ± 3.4 (ACW), *p* < 0.001	
DR	20	20	**↑**, 61.75 ± 19.84, *p* < 0.001	**↑**, 21.80± 10.44, *p* = 0.010	Ahsanuddin et al. 2023 [19]
	7	13	76.6 ± 15.7, *p* = 0.04 ^φ^	72.1 ± 15.9 (ACW) ^φ^	Elner et al. 2008 [28]
	12	24	69.7 ± 18.3, *p* = 0.002 ^φ^	63.2 ± 14.5 (ACW) gsu, *p* = 0.005 ^φ^	Field et al. 2008 [27]
NPDR	9	9	**↑**, 61.78 ± 18.51, *p* < 0.001	**↑**, 20.67 ± 7.62, *p* = 0.031	Ahsanuddin et al. 2023 [19]
Mild–Moderate NPDR	29	29	73.0 [68.5–95.0] ^¥^	0.85 [0.61–1.00] ^¥^	Chen et al. 2021 [23]
PDR	11	11	**↑**, 61.73 ± 21.76, *p* < 0.001	**↑**, 22.73 ±12.60, *p* = 0.046	Ahsanuddin et al. 2023 [19]
	25	25	73.0 [69.3, 88.8] ^¥^	0.90 [0.51, 1.20] ^¥^	Chen et al. 2021 [23]
DM (before Anti-VEGF)	8	8	433.9 ± 134.6	-	Romo et al. 2018 [24]
DM (after Anti-VEGF)	8	8	403.6 ± 143.3, *p* = 0.289 ^¶^	-	
CSR	10	10	**↑**, 53.80 ± 14.34, *p* = 0.001	**↑**, 18.70 ± 4.19, *p* = 0.037	Ahsanuddin et al. 2023 [19]
	3	3	40.4 ± 13.9 (calculated), *p* < 0.05 in all patients	-	Field et al. 2009 [26]
	1	2	79.5 ± 5.8 ^§^	68.0 ± 2.8 ^§^ (ACW)	Elner et al. 2008 [28]
Active CSR	6	6	**↑**, 60.00 ± 12.18, *p* < 0.001	18.00 ± 4.00	Ahsanuddin et al. 2023 [19]
Chronic Inactive CSR	4	6	44.50 ± 13.39, *p* = 0.074	19.75 ± 4.86	
Rod–Cone dystrophies (RP, US)	40	78	**↑**, *p* = 0.003	**↑**, *p* < 0.001	Russell et al. 2022 [22]
RP	1	2	50.5 ± 12.2 ^§^	48.0 ± 5.0 ^§^ (ACW)	Elner et al. 2008 [28]
STGD	16	31	**↑**, *p* < 0.001	**↑**, *p* < 0.001	Russell et al. 2022 [22]
BBS	2	4	**↑**, *p* < 0.001	**↑**, *p* = 0.011	
MELAS	3	6	*p* = 0.999	*p* = 0.999	
MT-ATP6	1	2	**↑**, *p* = 0.007	**↑**, *p* = 0.627	
Papilledema	6	12	**↑**, ≈24.78 ± 8.40 * (ACW), *p* = 0.018	**↑**, ≈25.88 ± 57 * (ACW), *p* = 0.039	Elner et al. 2008 [18]
ODD	94	157	20.6 ± 0.44, *p* = 0.148	-	Pujari et al. 2026 [12]

SD: Standard deviation, **↑**: higher than controls, *p*: *p*-value of the correlation of cases FPF versus controls or other group if specified (^φ^), ACW: Average curve width, ODD: Optic disc drusen, POAG: Primary open-angle glaucoma, GS/OHT: Glaucoma suspect/Ocular hypertension, AMD: Age-related macular degeneration, GA: Geographic atrophy, RVO: Retinal vessel occlusion, CRVO: Chronic RVO, BRVO: Branch RVO, DM: Diabetes mellitus, wo: without, DR: Diabetic retinopathy, NPDR: Non-proliferative DR, PDR: Proliferative DR, VEGF: Vascular endothelial growth factor, CSR Central serous retinopathy, RP: Retinitis pigmentosa, US: Usher syndrome, STGD: Stargardt disease, BBS: Bardet–Biedl syndrome, MELAS: Mitochondrial encephalomyopathy, lactic acidosis, and stroke-like episodes, MT-ATP6: Mitochondrially encoded ATP synthase membrane subunit 6. ^φ^ FPF signals from the higher severity disease subgroup compared to the lower severity disease subgroup; * value calculated from available data in the study results; ^§^ value estimated from available data in the study results; ^¶^ FPF signals compared within the same patient’s values before treatment; ^¥^ only the IQR range was available.

**Table 6:** Current FPF profiles categorized by disease. *p*-values are provided when cases are compared to controls, unless specified otherwise in the table.

Disease	ONH FPF Metrics		Reference
	Intensity (Mean ± SD gsu, *p*-Value)	Heterogeneity (Mean ± SD, *p*-Value)	
ODD	**↑**, 10.82 ± 0.36 dB, *p* < 0.001	-	Pujari et al. 2026 [12]
GS/OHT	91.4 ± 28.5, *p* = 0.207	19.2 ± 4.3	Caro et al. 2025 [14]
POAG	83.2 ± 33.8, *p* = 0.586	20.5 ± 4.8	Caro et al. 2025 [14]
	ONH rim: **↑**, 46.4 ± 27.9, *p* < 0.001 Temporal: **↑**, 68.5 ± 40.3 *p* = 0.001 Superior: **↑**, 32.8 ± 29.5 *p* < 0.001 Nasal: **↑**, 39.7 ± 27.1 *p* = 0.002 Inferior: **↑**, 45.9 ± 28.9 *p* = 0.001	-	Zhou et al. 2022 [21]
ONH disc: Baseline:	19.1 ± 3.7	-	Sun et al. 2022 [20]
Post-treatment:			
1 h	19.5 ± 3.3, *p* = 0.400 ^¶^	-	
1 mo	15.4 ± 5.8, *p* = 0.200 ^¶^	-	
ONH rim: Baseline:	12.7 ± 11.6	-	
Post-treatment:			
1 h	13.1 ± 9.3, *p* = 0.900 ^¶^	-	
1 mo	**↓** 10.5 ± 7.5, *p* = 0.040 ^¶^	-	

FPF: Flavoprotein fluorescence, ODD: Optic disc drusen, GS/OHT: Glaucoma suspect/Ocular hypertension, POAG: Primary open-angle glaucoma, ONH: Optic nerve head, SD: Standard deviation,, ↑: higher than controls, ↓: lower than controls, gsu: Grey scale units, dB: Decibels, h: Hour, mo: Month. ^¶^ FPF metrics compared within the same patient’s values before treatment.

## Data Availability

Data included in the present work are available from the corresponding author upon reasonable request.

## Supplementary Materials

The following supporting information can be downloaded at: https://www.mdpi.com/article/10.3390/jcm15103942/s1. Supplementary Table S1: Online databases and search equations used for the review. Supplementary Table S2: Preferred Reporting Items for Systematic reviews and Meta-Analyses extension for Scoping Reviews (PRISMA-ScR) Checklist. Supplementary Table S3: Demographic data collected from the studies. Supplementary Table S4: Correlation between clinical variables and FPF in healthy eyes. Supplementary Table S5: Correlations reported between FPF metrics and clinical variables by disease.

## Abbreviations

The following abbreviations are used in this manuscript:

AMD	Age-related macular degeneration
DR	Diabetic retinopathy
OCT	Optical coherence tomography
SLO	Scanning Laser Ophthalmoscopy
AOSLO	Adaptive Optics Scanning Laser Ophthalmoscopy
AONCO	Adaptive Optics Near-Confocal Ophthalmoscope
AO-RSO	Adaptive Optics Rolling Slit Ophthalmoscope
FAF	Fundus autofluorescence
FPF	Flavoprotein fluorescence
PRISMA	Preferred Reporting Items for Systematic Reviews and Meta-Analyses
PRISMA-ScR	PRISMA Extension for Scoping Reviews
MeSH	Medical Subject Headings
AUC	Area under the curve
R^2^	Coefficient of Determination from regression
PMID	PubMed Identifier
ABCA4	ATP Binding Cassette Subfamily A Member 4
GS	Glaucoma suspect
POAG	Primary open-angle glaucoma
ODD	Optic disc drusen
RVO	Retinal vein occlusion
CSR	Central serous retinopathy
BCVA	Best corrected visual acuity
IOP	Intraocular pressure
BGS	pre–Balance Goggles System
IRDs	Inherited retinal diseases
BBS	Bardet–Biedl syndrome
MELAS	Mitochondrial encephalomyopathy, lactic acidosis, and stroke-like episodes
MT-ATP6	Mitochondrial ATP synthase subunit 6 mutation
VEGF	Vascular endothelial growth factor
CSDME	Clinically significant diabetic macular edema
OHT	Ocular hypertension
DM	Diabetes mellitus
RP	Retinitis pigmentosa
OPL	Outer plexiform layer
INL	Inner nuclear layer
RGCs	Retinal ganglion cells
RNFL	Retinal nerve fiber layer
GCL	Ganglion cell layer
INLm	INL–OPL border
ONL	Outer nuclear layer
ELM	External limiting membrane
RPE	Retinal pigment epithelium
MTZ	Myelin transition zone
FMN	Flavin mononucleotide
FAD	Flavin adenine dinucleotide
NADH	Nicotinamide adenine dinucleotide hydrogen
ETF	Electron transfer flavoprotein
ΔΨm	Mitochondrial membrane potential
MI	Michigan
IR	Infrared
ONH	Optic nerve head
ROS	Reactive oxygen species
SD	Standard deviation
SI	Stress index
gsu	Gray-scale units
NADPH	Nicotinamide Adenine Dinucleotide Phosphate
NPDR	Non-proliferative diabetic retinopathy
PDR	Proliferative diabetic retinopathy
ETDRS	Early Treatment of Diabetic Retinopathy Study
cpRNFLT	Circumpapillary retinal nerve fiber layer
ACW	Average curve width
GA	Geographic atrophy
CRVO	Central retinal vein occlusion
BRVO	Branch retinal vein occlusion
wo	Without
mo	Month
USH	Usher syndrome
STGD	Stargardt disease
IPL	Inner plexiform layer
MD	Mean deviation
PSD	Pattern standard deviation
EDI-OCT	Enhanced Depth Imaging Optical Coherence Tomography
GCC	Ganglion cell complex
VF	Visual field
