# Supplementary material for: Quantitative Flavoprotein Fluorescence Parameters in Retinal and Optic Nerve Diseases: A Scoping Review
Source: J Clin Med. 2026 May 20;15(10):3942. doi: 10.3390/jcm15103942 (PMC13207118; doi:10.3390/jcm15103942)
Supplement: Supplementary file 1 [file jcm-15-03942-s001.zip › jcm-4298757-supplementary.pdf]

Supplementary material

Supplementary Table S1: Online databases and search equations used for the review.

<table>
<tr><td rowspan="12">MEDLINE (via Ovid, https://ovidsp.ovid.com)</td><td>1</td><td>exp Flavoproteins/</td><td>MeSH (Exploded)</td><td>62318</td></tr>
<tr><td>2</td><td>("FPF imaging" OR "redox imaging" OR "Mitochondrial fluorescence").tw.</td><td>Free-text (Title/Abstract)</td><td>230</td></tr>
<tr><td>3</td><td>#1 OR #2</td><td>Combined Technique Terms</td><td>1823947</td></tr>
<tr><td>4</td><td>exp Retinal Degeneration/</td><td>MeSH (Exploded)</td><td>55692</td></tr>
<tr><td>5</td><td>exp Optic Nerve Diseases/</td><td>MeSH (Exploded)</td><td>38718</td></tr>
<tr><td>6</td><td>exp Macular Degeneration/</td><td>MeSH (Exploded)</td><td>33355</td></tr>
<tr><td>7</td><td>exp Diabetic Retinopathy/</td><td>MeSH (Exploded)</td><td>32625</td></tr>
<tr><td>8</td><td>exp Glaucoma/</td><td>MeSH (Exploded)</td><td>63926</td></tr>
<tr><td>9</td><td>("Central serous chorioretinopathy" OR "Stargardt disease$" OR "Leber congenital amaurosis" OR "Leber hereditary optic neuropathy" or "optic atrophy$").tw.</td><td>Free-text (Title/Abstract)</td><td>11488</td></tr>
<tr><td>10</td><td>#4 or #5 or #6 or #7 or #8 or #9</td><td>Combined Disease Terms</td><td>183508</td></tr>
<tr><td>11-17</td><td colspan="3">Steps leading to the final concepts. Omitted for clarity.</td></tr>
<tr><td>18</td><td>((Flavoprotein$ adj3 (fluorescence or imaging or autofluorescence or redox)) or "FPF" or "FAD" or "redox imaging" or "Mitochondrial fluorescence" or "metabolic imaging").tw.</td><td>Comprehensive Free-text Technique Terms</td><td>15725</td></tr>
<tr><td></td><td>19</td><td>18 AND 10</td><td>Final Combined Search (Technique AND Disease)</td><td>36</td></tr>
<tr><td>Scopus (https://www.scopus.com/)</td><td colspan="4">((Flavoprotein* W/3 (fluorescence OR imaging OR autofluorescence OR redox)) OR "FPF" OR "FAD" OR "redox imaging" OR "Mitochondrial fluorescence" OR "metabolic imaging") AND (Retinal Degeneration* OR "Optic Nerve Disease*" OR "Macular Degeneration*" OR "Diabetic Retinopathy" OR Glaucoma OR "Central serous chorioretinopathy" OR "Stargardt disease*" OR "Leber congenital amaurosis" OR "Leber hereditary optic neuropathy" OR "optic atrophy*")</td></tr>
<tr><td>Web of Science (https://www.webofscience.com/)</td><td colspan="4">(TS = (Flavoprotein* NEAR/3 (fluorescence OR imaging OR autofluorescence OR redox) OR "FPF" OR "FAD" OR "redox imaging" OR "Mitochondrial fluorescence" OR "metabolic imaging")) AND (</td></tr>
</table>

https://doi.org/10.3390/xxxxx

	TS = (Retinal Degeneration* OR "Optic Nerve Disease*" OR "Macular Degeneration*" OR "Diabetic Retinopathy" OR Glaucoma OR "Central serous chorioretinopathy" OR "Stargardt disease*" OR "Leber congenital amaurosis" OR "Leber hereditary optic neuropathy" OR "optic atrophy*"))
**Cochrane** (https://www.cochrane.org/)	(Flavoprotein* near/3 (fluorescence or imaging or autofluorescence or redox)) or FPF or FAD or "redox imaging" or "Mitochondrial fluorescence" or "metabolic imaging" in Title Abstract Keyword AND (Retinal near/3 Degeneration*) or ("Optic Nerve" near/3 Disease*) or ("Macular" near/3 Degeneration*) or ("Diabetic" near/3 Retinopathy) or Glaucoma or ("Central serous" near/3 chorioretinopathy) or ("Stargardt" near/3 disease*) or ("Leber congenital" near/3 amaurosis) or ("Leber hereditary" near/3 "optic neuropathy") or ("optic" near/3 atrophy*) in Title Abstract Keyword - (Word variations have been searched

Supplementary Table S2: Preferred Reporting Items for Systematic reviews and Meta-Analyses extension for Scoping Reviews (PRISMA-ScR) Checklist [17].

SECTION	ITEM	PRISMA-ScR CHECKLIST ITEM	REPORTED ON PAGE #
**TITLE**			
Title	1	Identify the report as a scoping review.	1
**ABSTRACT**			
Structured summary	2	Provide a structured summary that includes (as applicable): background, objectives, eligibility criteria, sources of evidence, charting methods, results, and conclusions that relate to the review questions and objectives.	1
**INTRODUCTION**			
Rationale	3	Describe the rationale for the review in the context of what is already known. Explain why the review questions/objectives lend themselves to a scoping review approach.	1-2
Objectives	4	Provide an explicit statement of the questions and objectives being addressed with reference to their key elements (e.g., population or participants, concepts, and context) or other relevant key elements used to conceptualize the review questions and/or objectives.	2
**METHODS**			
Protocol and registration	5	Indicate whether a review protocol exists; state if and where it can be accessed (e.g., a Web address); and if available, provide registration information, including the registration number.	2-3
Eligibility criteria	6	Specify characteristics of the sources of evidence used as eligibility criteria (e.g., years considered, language, and publication status), and provide a rationale.	3
Information sources*	7	Describe all information sources in the search (e.g., databases with dates of coverage and contact with authors to identify additional sources), as well as the date the most recent search was executed.	2

SECTION	ITEM	PRISMA-ScR CHECKLIST ITEM	REPORTED ON PAGE #
Search	8	Present the full electronic search strategy for at least 1 database, including any limits used, such that it could be repeated.	2-3
Selection of sources of evidence†	9	State the process for selecting sources of evidence (i.e., screening and eligibility) included in the scoping review.	2-3
Data charting process‡	10	Describe the methods of charting data from the included sources of evidence (e.g., calibrated forms or forms that have been tested by the team before their use, and whether data charting was done independently or in duplicate) and any processes for obtaining and confirming data from investigators.	3-4
Data items	11	List and define all variables for which data were sought and any assumptions and simplifications made.	3-4
Critical appraisal of individual sources of evidence§	12	If done, provide a rationale for conducting a critical appraisal of included sources of evidence; describe the methods used and how this information was used in any data synthesis (if appropriate).	-
Synthesis of results	13	Describe the methods of handling and summarizing the data that were charted.	4
**RESULTS**			
Selection of sources of evidence	14	Give numbers of sources of evidence screened, assessed for eligibility, and included in the review, with reasons for exclusions at each stage, ideally using a flow diagram.	4
Characteristics of sources of evidence	15	For each source of evidence, present characteristics for which data were charted and provide the citations.	4-6
Critical appraisal within sources of evidence	16	If done, present data on critical appraisal of included sources of evidence (see item 12).	-
Results of individual sources of evidence	17	For each included source of evidence, present the relevant data that were charted that relate to the review questions and objectives.	7-11
Synthesis of results	18	Summarize and/or present the charting results as they relate to the review questions and objectives.	11-22
**DISCUSSION**			
Summary of evidence	19	Summarize the main results (including an overview of concepts, themes, and types of evidence available), link to the review questions and objectives, and consider the relevance to key groups.	22
Limitations	20	Discuss the limitations of the scoping review process.	22-23
Conclusions	21	Provide a general interpretation of the results with respect to the review questions and objectives, as well as potential implications and/or next steps.	23
**FUNDING**			
Funding	22	Describe sources of funding for the included sources of evidence, as well as sources of funding for the scoping review. Describe the role of the funders of the scoping review.	24

JBI = Joanna Briggs Institute; PRISMA-ScR = Preferred Reporting Items for Systematic reviews and Meta-Analyses extension for Scoping Reviews.

* Where *sources of evidence* (see second footnote) are compiled from, such as bibliographic databases, social media platforms, and Web sites.

† A more inclusive/heterogeneous term used to account for the different types of evidence or data sources (e.g., quantitative and/or qualitative research, expert opinion, and policy documents) that may be eligible in a scoping review as opposed to only studies. This is not to be confused with *information sources* (see first footnote).

‡ The frameworks by Arksey and O'Malley (6) and Levac and colleagues (7) and the JBI guidance (4, 5) refer to the process of data extraction in a scoping review as data charting.

§ The process of systematically examining research evidence to assess its validity, results, and relevance before using it to inform a decision. This term is used for items 12 and 19 instead of "risk of bias" (which is more applicable to systematic reviews of interventions) to include and acknowledge the various sources of evidence that may be used in a scoping review (e.g., quantitative and/or qualitative research, expert opinion, and policy document).

Supplementary Table S3*:* Demographic data collected from the studies.

PMID	Author (year)	Subjects (total)	Patients			Controls			Eyes			
			Total	females	males	Total	females	males	1 or 2 included	Total	patients	controls
41260483	Pujari et al. (2026) [12]	147	94	67	27	53	28	25	2, independent	226	157	69
40767444	Merle et al. (2025) [6]	36	36	23	13	0	–	–	2, independent	72	72	0
40631437	Kim et al. (2025) [15]	0	0	–	–	75	28	47	2, mean	147	0	147
39808681	Caro et al. (2025) [14]	95	70	39	31	25	17	8	1, independent	95	70	25
36626211	Muste et al. (2023) [13]	260	130	72	58	130	72	58	2, independent	390	195	195
38983095	Ahsanuddin et al. (2023) [19]	88	67	42	25	21	10	11	1, independent	88	67	21
35696700	Sun et al. (2022) [20]	8	8	3	5	0	0	0	1, independent	8	8	0
34968754	Zhou et al. (2022) [21]	50	30	15	15	20	9	11	2, independent	86	50	36
36384402	Russell et al. (2022) [22]	157	61	40	21	96	38	58	2, independent	242	121	121
32709959	Chen et al. (2021) [23]	268	117	60	57	151	69	82	1, independent	268	117	151
29750714	Geyman et al. (2018) [16]	43	27	–	–	16	–	–	2, independent	86	54	32
30159113	Romo et al. (2018) [24]	8	8	2	6	0	0	0	1, independent	8	8	0
22822904	Field et al. (2012) [25]	8	5	–	–	3	–	–	2, independent	9	6	3

19491721	Field et al. (2009) [26]	6	3	0	3	3	–	–	2, independent	9	3	6
18625939	Field et al. (2008) [27]	42	21	–	–	21	–	–	2, independent	84	42	42
18268219	Elner et al. (2008) [28]	36	17	–	–	19	–	–	2, independent	72	34	38
19277237	Elner et al. (2008) [18]	12	6	6	0	6	6	0	2, independent	24	12	12

Supplementary Table S4: Correlation between clinical variables and FPF in healthy eyes**.**

	Findings and correlations	Reference
Age	- ↑FPF with ↑age: macular (r = 0.50, $p < 0.001$) and ONH (r = 0.70, $p < 0.001$)	Kim et al. (2025) [15]
	- Non-significant correlation with FPF ($p > 0.2$)	Merle et al. (2025) [6]
	- Non-significant correlation, FPF stable from 1st to 7th decade	Pujari et al. (2026) [12]
	- ↑FPF with - age: +0.21 (0.08 to 0.35) dB per year, $p = 0.002$	Muste et al. (2023) [13]
	- Non-significant correlation with FPF	Zhou et al. (2022) [21]
	- ↑FPF intensity with -age: β= +1.40, CI: +1.26 to +1.55, $p < 0.001$<br>- ↑FPF intensity and heterogeneity with age, $p< 0.001$	Chen et al. (2021) [23]
	- ↑FPF increases with ↑age ($p < 0.05$)	Elner et al. (2008) [28]
OCT	- ↑FPF with:<br>o TRT (r = –0.49, $p < 0.001$)<br>o RNFL (r = –0.48, $p < 0.001$)<br>o GCIPL (r = –0.59, $p < 0.001$)<br>o OPL–EZ region (r = –0.24, $p = 0.041$)<br>o ReP layer (r = –0.34, $p = 0.003$)	Kim et al. (2025) [15]
	- Non-significant correlation of FPF with RNFL thickness, BMO-MRW, or neuroretinal rim area	Zhou et al. (2022) [21]
BCVA	- ↑macular FPF intensity with ↓BCVA (r=0.595, CI: 0.362 – 0.666, $p<0.001$)	Ahsanuddin et al. (2023) [19]

	- ↑macular FPF heterogeneity with ↓BCVA (r=0.306, CI: –0.089 – 0.323, *p=0.004*)	
	- FPF signals were not significant predictors of visual acuity	Russell et al. (2022) [22]
VF	- FPF has non-significant correlation with VF MD (r = 0.06, *p = 0.79*) or PSD r = –0.05 (*p = 0.84*)	Zhou et al. (2022) [21]
Pseudophakia	- ↓FPF (β: −2.90 CI: –5.12 to –0.68, *p=0.011*)	Muste et al. (2023) [13]
Gender	- Males have slightly ↓FPF intensity (β = –1.93, p = *0.049*).	Muste et al. (2023) [13]
	- Females have ↑FPF intensity (β: +6.46, 95 % CI: +2.98 to +9.93, *p < 0.001)*	Chen et al. (2021) [23]
Black	- ↓FPF intensity (β = –4.05, p = 0.024).	Muste et al. (2023) [13]
Smoking	- ↓FPF in former (β = –6.24, *p=0.002*) or never (β = –6.7, *p=0.001*) smokers	Muste et al. (2023) [13]
	- Non-significant correlation with FPF intensity, *p > 0.1*	Chen et al. (2021) [23]
IOP	- Non-significant correlation with FPF intensity (β= –0.17, CI: –0.74 to +0.40, *p=0.56*)	Chen et al. (2021) [23]

FPF: Flavoprotein fluorescence, ↑: higher, ↓: lower, ONH: Optic nerve head, r: Correlation coefficient, TRT: Total retinal thickness, RNFL: Retinal nerve fiber layer, GCIPL: Ganglion cell-inner plexiform layer, BMO-MRW: Bruch's Membrane Opening – Minimum Rim Width, IOP: Intraocular pressure, CI: Confidence interval, BCVA: Best corrected visual acuity, dB: Decibels. ReP: Retinal photoreceptor, β: Regression coefficient.

Supplementary Table S5: Correlations reported between FPF metrics and clinical variables by disease.

Disease	Age (mean ± SD)	OCT	FAF	BCVA	VF	Reference

Retinal dystrophies	39.7	Qualitative association, the FPF signal localizes to regions of outer retinal degeneration.	Qualitative association, the FPF signal is present in both hyperfluorescent regions and hypofluorescent (atrophic) regions	- FPF intensity in RD vs visual acuity in logMAR (Rod-Cone Dystrophy 0.79 ± 0.58, SD 0.96 ± 0.67, BBS 0.95 ± 0.66, MT-ATP6 Mutation 0.49 ± 0.39, MELAS 0.17 ± 0.14): β = +0.12, *p = 0.481*,<br>FPF heterogeneity vs BCVA in logMAR: β = +2.99, *p = 0.565*	NA	Russell et al. 2022 [22]
RP	36.0	NA	NA	NA	↑FPF in the eyes with ring scotoma	Elner et al. 2008 [28]
STGD	34.5 ± 17.33	**Lesion type** / **FAF-OCT-FPF correlation**<br>**Type A:** ↑FPF – ↑FAF – OCT: subretinal lesions in OCT (likely due to lipofuscin).<br>**Type B:** ↑FPF – normal or ↓FAF – OCT: atrophy of the outer retinal layers with hyperreflective material above the RPC.<br>**Type C:** ↑FPF – ↑FAF – OCT: thickening and blurring of the ellipsoid zone and interdigitation zone above intact RPE.<br>**Type D:** Slightly ↑FPF – slightly ↑FAF – OCT: slightly irregular configuration of the outer retinal layers, along with a granular appearance of the RPE.<br>**Type E:** ↓FPF – ↓FAF – OCT: complete atrophy of the outer retinal layers and the RPE.<br>**Foveal sparing:** Near-normal FAF – OCT: preserved outer retinal layers.		1.03 logMAR	NA	Merle et al. 2025 [6]
		**Lesion categories based on which retinal area produced the autofluorescence:**<br>**Category 1 (57%):** Maximal correlation in the outer retina – photoreceptor outer segments.<br>**Category 2 (22%):** Correlation with both the inner and outer retina, positive correlation at the bipolar cell level, and at the level of photoreceptors.<br>**Category 3 (21%):** No strong correlation was found with any layer.				
	NA	NA	- Stratified retinal regions into 3 groups based on FAF features (normal,	No correlation between BCVA and FPF intensity, nor heterogeneity	NA	Russell et al. 2022 [22]

			hyperautofluorescent, and hypoautofluorescent/atrophic).<br>- Significant differences in FPF intensity across these regions, including elevations in areas without a corresponding FAF signal.<br>- FAF imaging alone did not reveal these metabolic differences.			
ODD	38.50	ODD ONH with VF loss:<br>- ↑FPF intensity with ↓RNFL thickness (79.4 ± 13.2 μm, r = −0.78, p < 0.001)<br>- -FPF intensity with ¯GCC thickness (78.6 ± 8.7 μm, r = −0.62, p = 0.009)<br>ODD ONH without VF loss:<br>- Non-significant FPF intensity change with ¯RNFL thickness or with ¯GCC thickness.	NA	0.08 ± 0.02 logMAR	↑FPF intensity (non-linear) with -VF MD (−5.91 ± 0.51 dB, R^2 = 0.45, p < 0.001, inflection point at MD ≈ −2.11 dB)	Pujari et al. 2026 [12]
GS	67.8 ± 10.3	Non-significant ONH FPF intensity correlations:<br>- RNFL thickness 88.2 ± 10.7 μm, r = 0.00, p = 0.990*<br>- Total macular thickness 281.1 ± 15.0 μm, r=-0.014, p=0.967*<br>- GCIPL thickness 55.07 ± 5.82 μm, r = –0.30, p = 0.300*	NA	Non-significant FPF intensity correlation with visual acuity (0.090 ± 0.109 logMAR, r = 0.16, p = 0.610*)	Non-significant ONH FPF intensity correlations:<br>- VF MD -1.31 ± 3.11 dB, r = 0.16, p = 0.610*<br>- VF PSD: 2.73 ± 1.45 dB r = 0.02, p = 0.950*<br>- VFI 97.6 ± 2.9 dB, r = 0.14, p = 0.660*	Caro et al. 2025 [14]
	57 ± 6.0	FPF/RGC+ thickness ratio:<br>- GS/OHT: - 4.8 ± 1.5 vs controls, p < 0.01	NA	NA	NA	Geyman et al. 2018 [16]
OAG	67.9 ± 11.6	ONH FPF intensity NS correlations:<br>- Temporal RNFL thickness 69.2 ± 14.2 μm, r: 0.080, p=0.571<br>- Total Macula thickness 272.9 μm ± 16.7. r: 0.169, p=0.227<br>- GCIPL thickness 50.32 ± 6.00 μm, r: 0.223, p=0.108	NA	- Lower visual acuity (0.083 ± 0.111 logMAR*) with -FPF intensity: r = –0.52, p = 0.045<br>- Non-significant correlation in visual acuity with FPF heterogeneity vs: r = –0.01, p = 0.960	ONH FPF intensity NS correlations:<br>- VF MD -5.36 ± 6.12 dB, r: 0.209, p=0.130<br>- VF PSD 4.87 ± 3.41 dB, r: –0.129, p=0.351	Caro et al. 2025 [14]

https://doi.org/10.3390/xxxxx

					- VFI: 87.4 ± 14.8 dB, r: 0.168, p=0.225	
	52.80	NA	RNFL thickness increased slightly from 69.5 ± 14.2 µm to 72.0 ± 13.7 µm (p = 0.10)	- 20/40 (62.5%)<br>- 20/40-20/60 (37.5%)	- Baseline VF MD = -4.41 dB (SD 4)<br>- Baseline VFI = 88 % (SD 11)	Sun et al. 2022 [20]
	57 ± 8.2	- -FPF intensity with ¯global circumpapillary RNFL thickness (β = −0.388 ± 0.111, p = 0.001)<br>- -FPF with ¯superior RNFL thickness: β = −0.203 ± 0.095, p = 0.035.<br>- -FPF with ¯nasal RNFL thickness: β = −0.341 ± 0.136, p = 0.015.<br>- - FPF with ¯inferior RNFL thickness: β = −0.283 ± 0.099, p = 0.006.<br>- Non-significant correlation of FPF and temporal RNFL thickness: β = +0.185 ± 0.361, p = 0.611.	NA	<20/200	- -FPF intensity associated with ¯VF MD (β = -0.996, CI -1.445 to -0.548, p < 0.001)<br>- -FPF intensity associated with -localized VF loss PSD (β = +2.860 ± 0.912, p = 0.003)	Zhou et al. 2022 [21]
	59±9 (**-** FPF with age, *p < 0.001*)	FPF/RGC+ thickness ratio:<br>- - 5.4 ± 2.1 vs controls, p < 0.001<br>FPF correlations:<br>- cpRNFL thickness: 71 ± 16 µm, β = 0.04, p > 0.05<br>- RGC+ thickness: 75 ± 13 µm, β = 0.61, p > 0.05	Automatic segmentation of ganglion cell and inner plexiform layers	>20/200	FPF intensity correlation:<br>- VF MD: −8.9 ± 8.6, β = 0.92, p > 0.05<br>- PSD: β = 0.94, p > 0.05	Geyman et al. 2018 [16]

RVO						
	59 ± 10	NA	NA	¯visual acuity (BCVA = 0.38 ± 0.32 logMAR) with **-** FPF intensity and FPF, *$p < 0.05$* (comparison across all diseases studied by the author)	NA	Ahsanuddin et al. 2023 [19]
DM						
	59 ± 11.25	NA	NA	DR: ¯visual acuity (BCVA = 0.25 ± 0.18 logMAR) with **-** FPF intensity (r ≈0.55–0.60, *$p<0.001$*) and **-** FPF heterogeneity (r = 0.306, *$p = 0.004$*)	NA	Ahsanuddin et al. 2023 [19]
	63.5, **-**FPF intensity with **-**age (+1.25 units/year, *$p<$ 0.001*) and **-**heterogeneity: (+0.02 units/year, *$p < 0.001$)*	NA	CST: 247.5 [236.0, 291.0]	DM: ¯visual acuity (BCVA = 80.2 [76.2–85.0] ETDRS) **-** FPF heterogeneity (r = –13.23, *$p<0.001$*)		Chen et al. 2021 [23]
	58.5 ± 9.01*	NA	FPF intensity vs OCT CMT: r = 0.617, *p = 0.192*	Percentual changes in FPF intensity vs in BCVA improvement, after treatment with Anti-VEGF, were correlated (r = 0.982, *p=0.000015)*	NA	Romo et al. 2018 [24]
	44.8 ± 10.0	NA	NA	Not reported	NA	Field et al. 2008 [27]
	61.0 ± 4.6	NA	NA	≥ 20/40 (one eye 20/200)	NA	Elner et al. 2008 [28]

AMD						
	73.10	- Retinal thickness (central subfield) vs FPF Intensity (r ≈ -0.12, *p = 0.320*).<br>- Outer retinal integrity (qualitative) vs FPF intensity (qualitative association): areas of EZ/RPE disruption show higher FPF signal (qualitative match on end face images)	FAF pattern (granularity) vs FPF heterogeneity (qualitative match): regions of granular FAF often exhibit elevated FPF heterogeneity, but not a 1:1 correlation.	¯visual acuity (72.4 [70.1–74.8] ETDRS letters) with:<br>- **-**FPF intensity (≈ 4-letter loss per 10 units FPF increase, β = – 4.09, 95% CI – 7.47 to – 0.70, *p = 0.018*)<br>- **-**FPF heterogeneity (β = – 0.14, 95% CI – 0.26 to – 0.02, *p = 0.024*)	NA	Muste et al. 2023 [13]
	70 ± 15	NA	NA	Exudative AMD: ¯visual acuity (0.32 ± 0.19 logMAR) with **-**FPF intensity and FPF heterogeneity, *p < 0.05* (comparison across all diseases studied by the author)	NA	Ahsanuddin et al. 2023 [19]
	76.5 ± 4.3	NA	NA	NA	NA	Field et al. 2012 [25]
	77	NA	NA	Qualitative only, ↓visual acuity with **-**FPF in ARMD (20/400 OS, 20/50 OD)	NA	Elner et al. 2008 [28]
CSR	54 ± 11.75		NA	¯visual acuity (BCVA = 0.18 ± 0.15 logMAR) with **-**FPF intensity and heterogeneity, *p < 0.05* (comparison across all diseases studied by the author)		Ahsanuddin et al. 2023 [19]
	35.67 ± 6.02	**-**FPF colocalized with lesions (qualitative)	NA	Patient 1 (R 20/20, L 20/20); Patient 2 (R 20/251, L 20/20); Patient 3 (R 20/15, L 20/30)	NA	Field et al. 2009 [26]
	NA	NA	**-**FPF matched **-**FAF areas	20/20 OU	NA	Elner et al. 2008 [28]
IIH	36.3 ± 5.9	NA	NA	20/30 OU or better	NA	Elner et al. 2008 [18]

SD: standard deviation; OCT: optical coherence tomography; FAF: fundus autofluorescence; FPF: flavoprotein fluorescence; BCVA: best-corrected visual acuity; VF: visual field; RD: retinal dystrophies; RP: retinitis pigmentosa; STGD: Stargardt disease; BBS: Bardet–Biedl syndrome; MT-ATP6: mitochondrially encoded ATP synthase membrane subunit 6; MELAS: mitochondrial encephalomyopathy, lactic acidosis, and stroke-like episodes; ODD: optic disc drusen; ONH: optic nerve head; GS: glaucoma suspect; OHT: ocular hypertension; OAG: open-angle glaucoma; RVO: retinal vein occlusion; DM: diabetes mellitus; DR: diabetic retinopathy; AMD: age-related macular degeneration; CSR: central serous chorioretinopathy; IIH: idiopathic intracranial hypertension; RNFL: retinal nerve fiber layer; GCC: ganglion cell complex; GCIPL: ganglion cell–inner plexiform layer; RGC+: retinal ganglion cell complex; cpRNFL: circumpapillary retinal nerve fiber layer; EZ: ellipsoid zone; RPE: retinal pigment epithelium; CST: central subfield thickness; CMT: central macular thickness; VEGF: vascular endothelial growth factor; ETDRS: Early Treatment Diabetic Retinopathy Study; CI: confidence interval; β: regression coefficient; r: correlation coefficient; R^2: coefficient of determination; MD: mean deviation; dB: decibels; PSD: pattern standard deviation; VFI: visual field index; R: right eye; L: left eye; OD: right eye; OS: left eye; OU: both eyes; NA: not applicable; ↑: higher; ↓: lower. * Calculated from the available study data.

https://doi.org/10.3390/xxxxx
